# Supplementary material for: Breakage fusion bridge cycles drive high oncogene number with moderate intratumoural heterogeneity
Source: Nat Commun. 2025 Feb 10;16:1497. doi: 10.1038/s41467-025-56670-8 (PMC11811125; doi:10.1038/s41467-025-56670-8)
Supplement: Supplementary file 1 — Supplementary Information [file 41467_2025_56670_MOESM1_ESM.pdf]

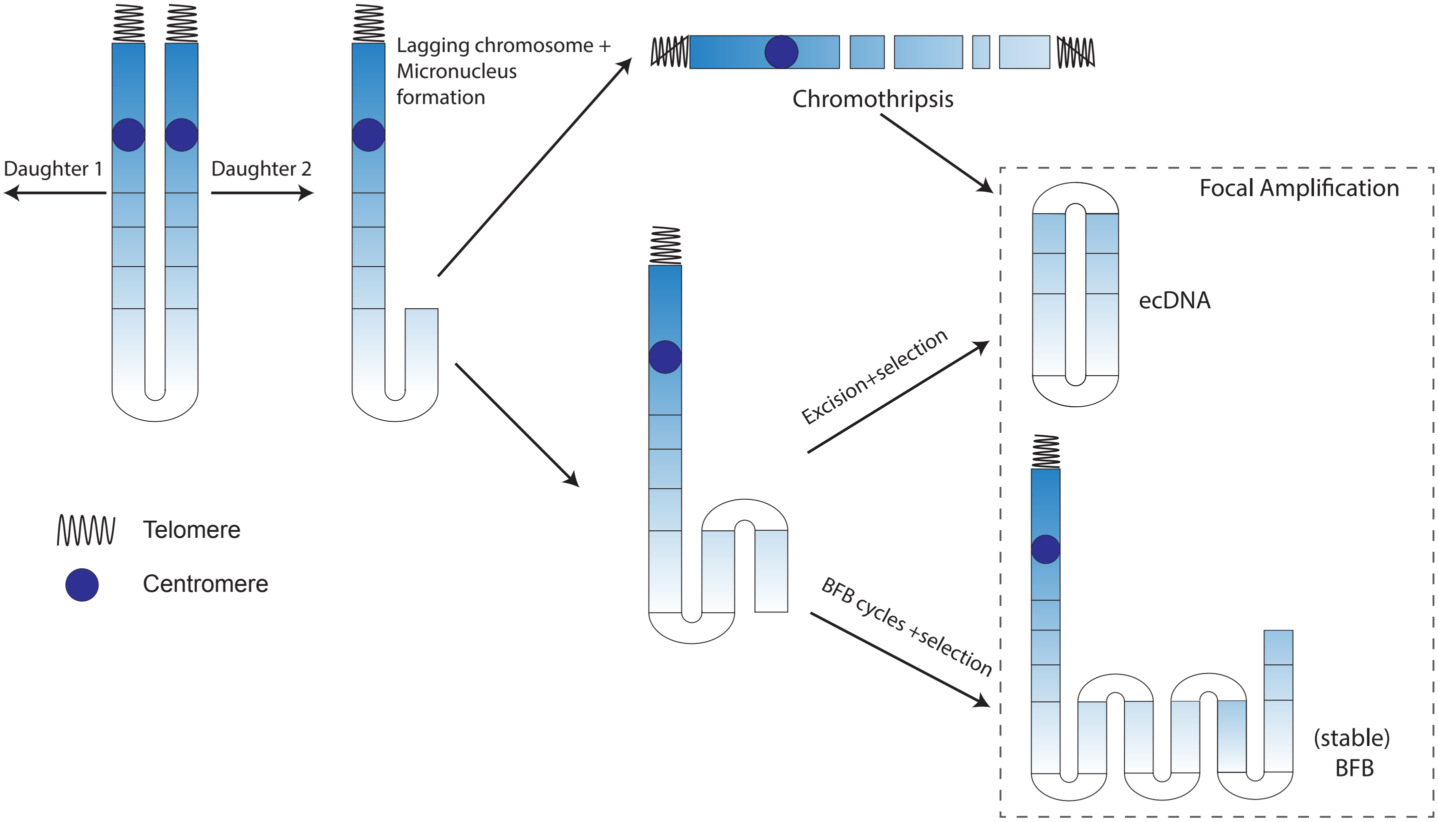

Supplementary Figure 1: Illustration of the initial BFB cycles outcome. BFB cycle can lead to ecDNA, chromothripsis, and also stable BFB cycles. Stable BFB cycles that manifest as an HSR on the native chromosome.

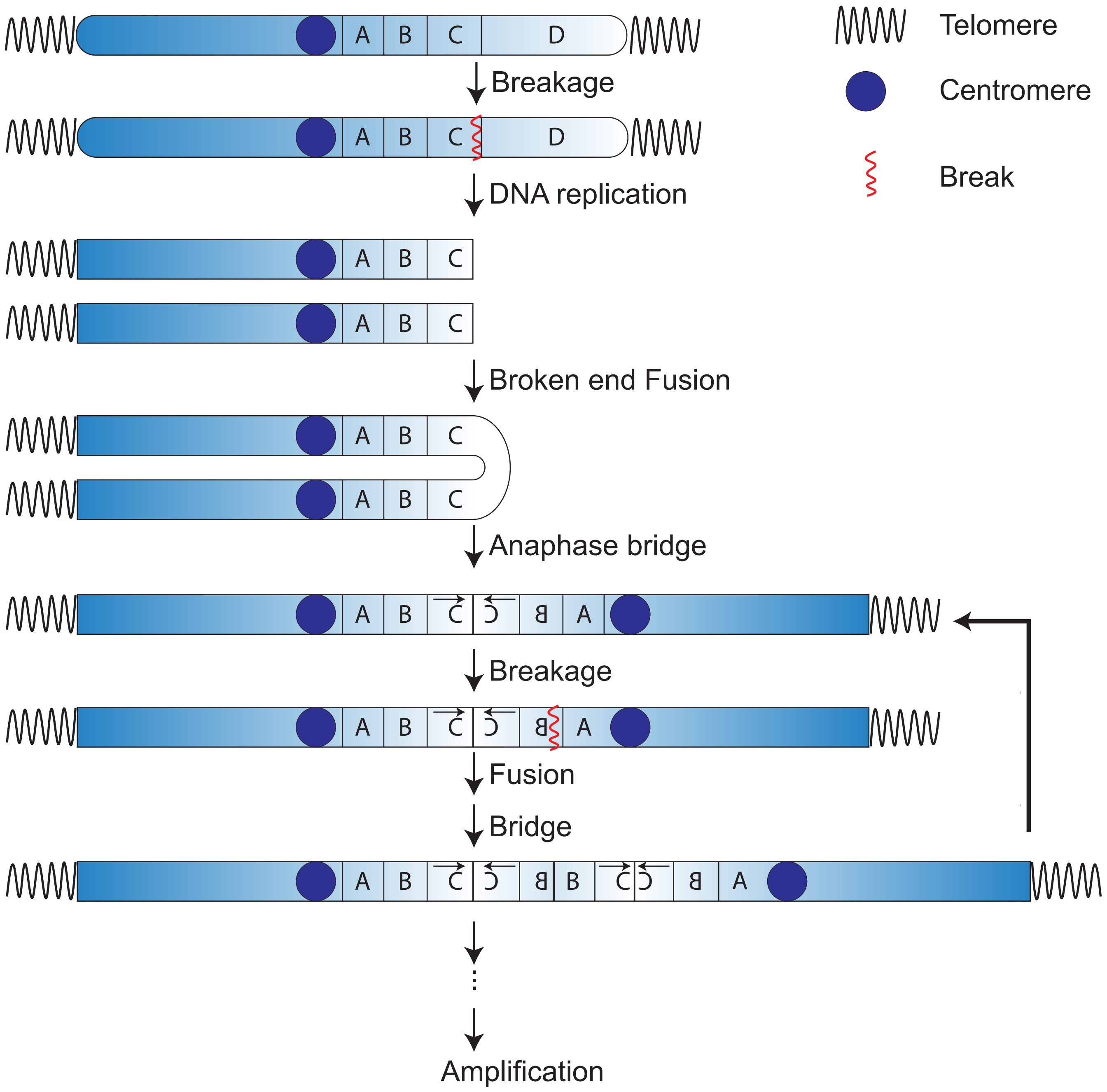

Supplementary Figure 2: Illustration of the Breakage-Fusion-Bridge (BFB) Mechanism leading to Focal Copy Number Amplification. The BFB mechanism starts with a telomeric break (loss of segment D) that is stabilized by the formation of an anaphase bridge between sister chromatids. Unequal breakage during cytokinesis leads to an inverted duplication genotype ABC→ ABCCB, with a broken end, leading to multiple cycles of bridge formation and breakage until the telomere is recapped, resulting in stable focal amplification with an excess of foldbacks and ladder-like amplification structure.

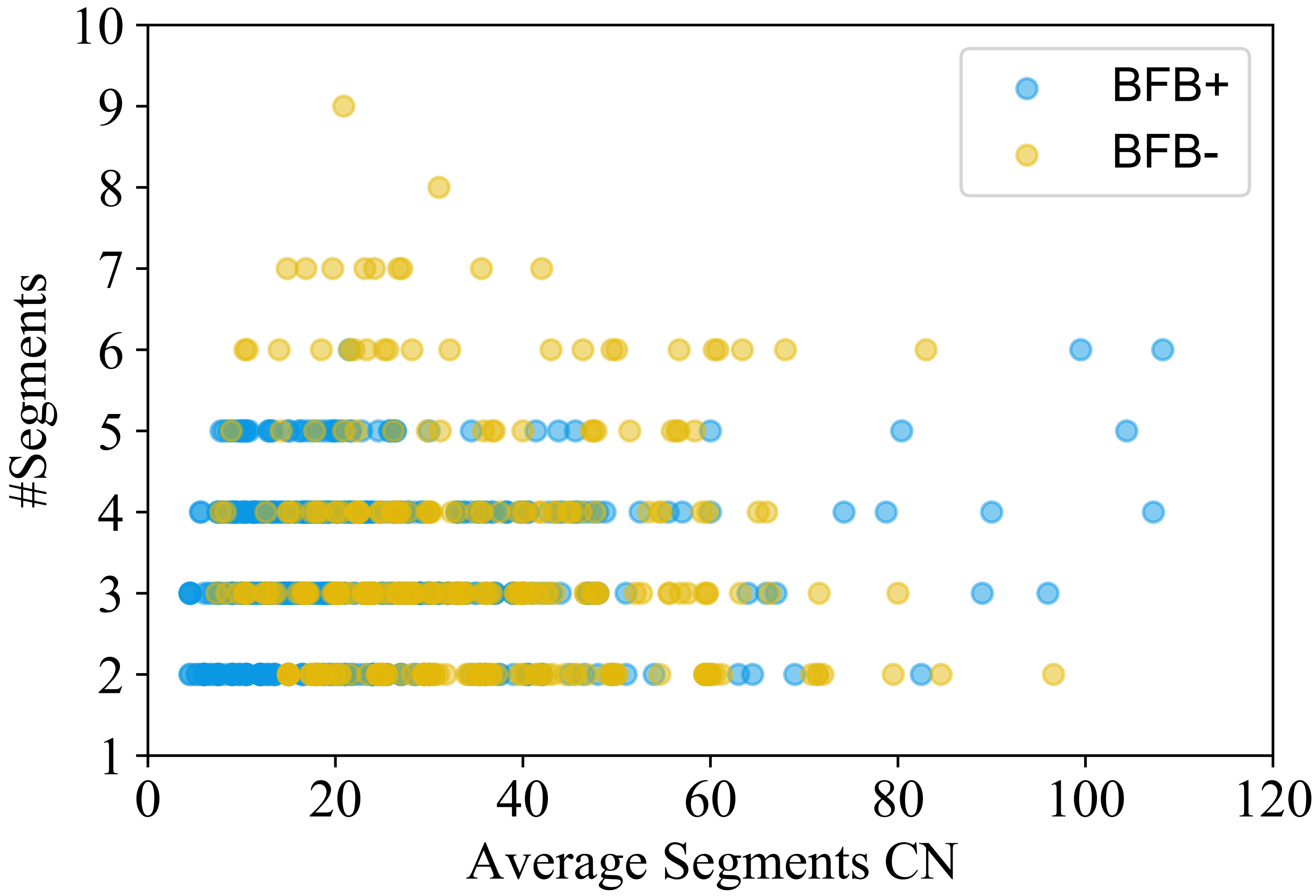

Supplementary Figure 3: Distribution of Simulated Cases over the Average Segments Copy Number and Number of Segments. A scatter plot of the BFB(+) and BFB(-) simulations, characterized by their average segment copy number and the number of segments showing little bias between positive and negative examples. Consistent with prior knowledge, ecDNAs, part of the BFB(-) examples, have higher CN values. Source data are provided as a Source Data file.

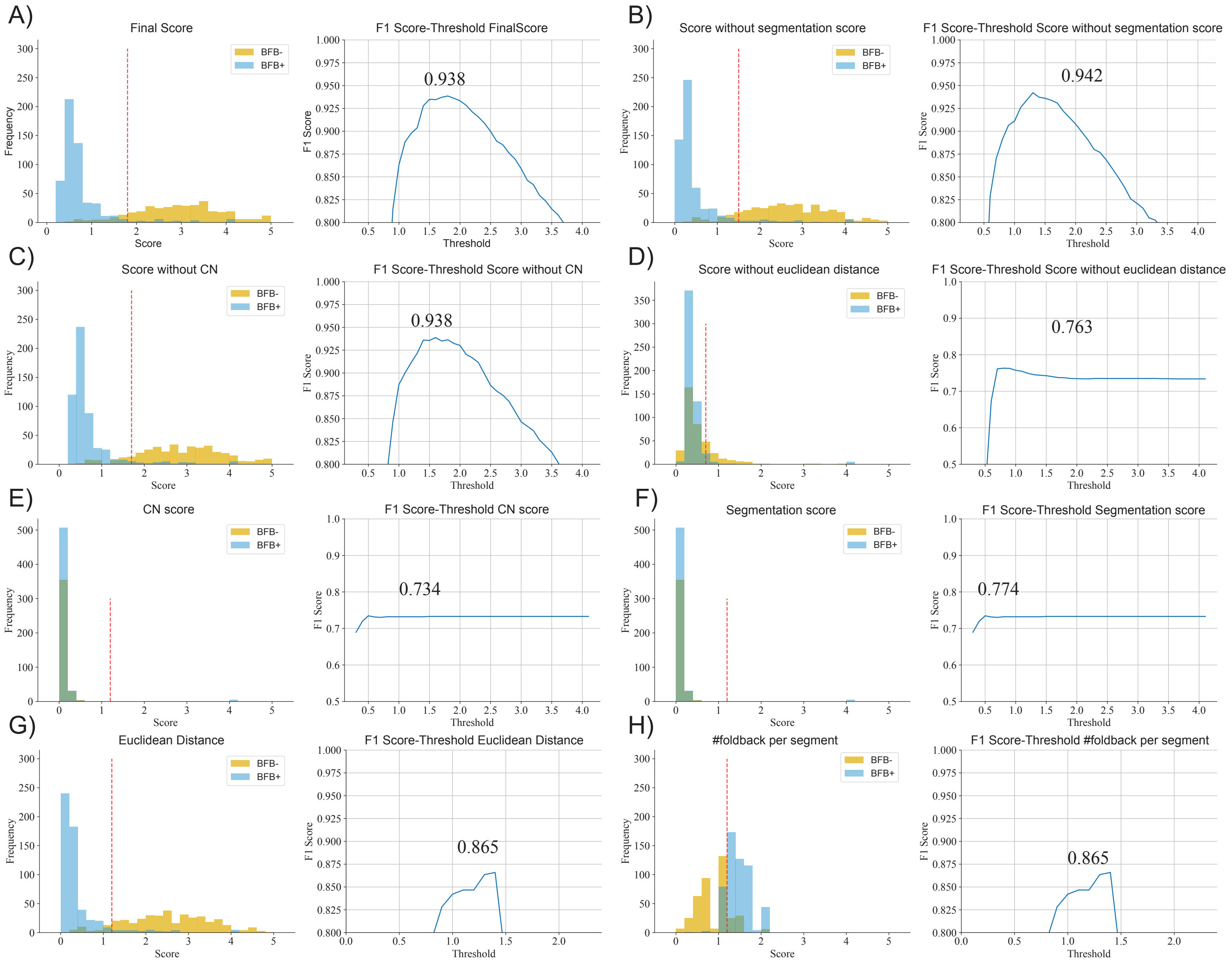

Supplementary Figure 4: Evaluating the Scoring Metrics for Distinguishing BFB Positive and Negative Cases. The plot shows the score distribution of 595 BFB(+) and the 389 BFB(-) cases remaining after discarding BFB(-) cases that did not meet the filtering threshold. OM2BFB scoring has 3 components: a copy number score, a segmentation score and a (Euclidean) distance from a candidate BFB. (A) The distribution achieved by the final score, and its performance in terms of the F1 value. (B-D) The distribution and performance after removing one of the 3 components of the score function. (E-H) The distribution and performance after retaining only one of the 3 components of the score function. The results suggest that each component distinctively contributes to improving the performance. Source data are provided as a Source Data file.

False Negative

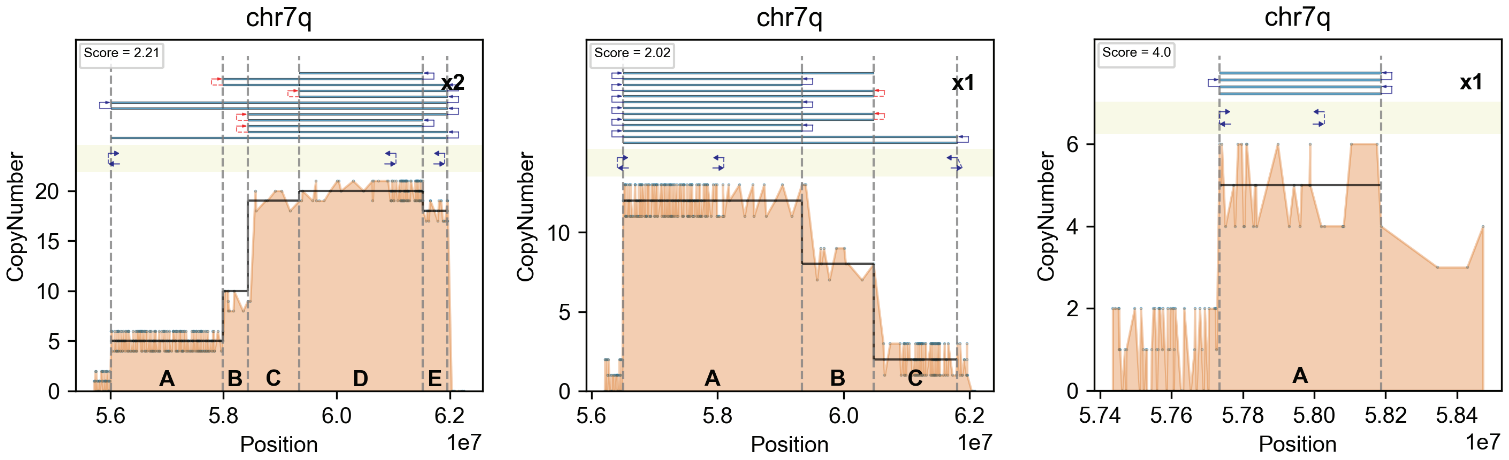

False Positive

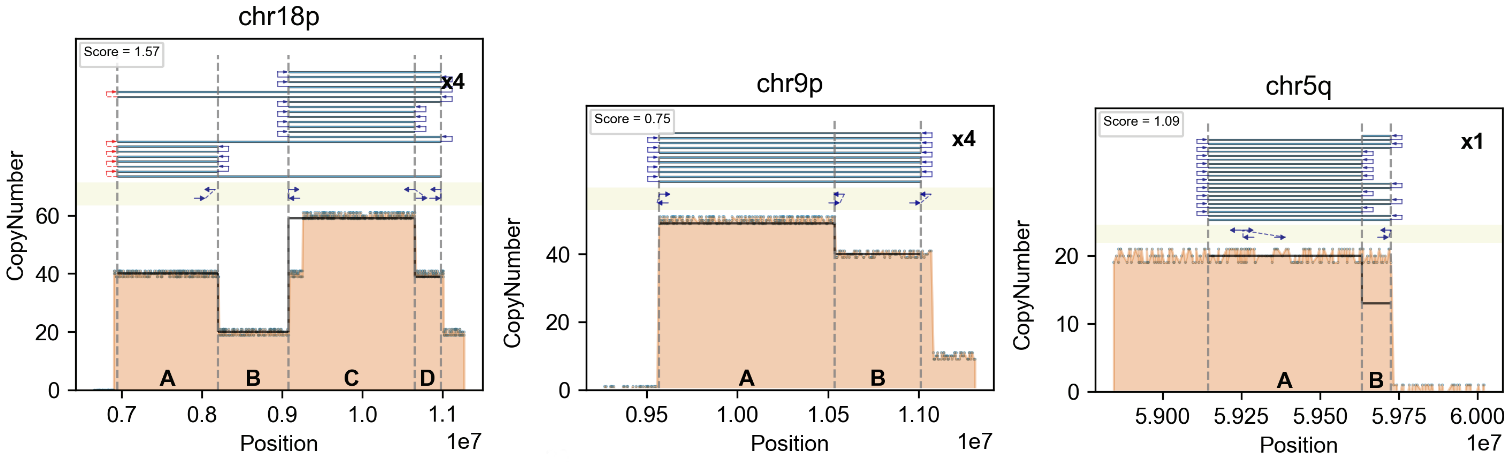

Supplementary Figure 5: Exemplars of false OM2BFB Predictions in simulated cases. (Top row) 'False-negative' cases that are BFB(+) but were erroneously predicted as BFB(-). The absence of foldback reads (represented in red) reduces the overall score (Left and Middle). Additionally, OM2BFB filters out instances where only one segment remains after segmentation, as those might correspond to ecDNA that originated as BFB (Right). (Bottom row) 'False-positive' cases. Non BFB based inverted duplications followed by ecDNA formation sometimes leads to a BFB-like signature. When the number of segments with foldbacks is large (e.g. 4), the absence of 1-2 foldbacks does not significantly impact the score, resulting in some false positive predictions.

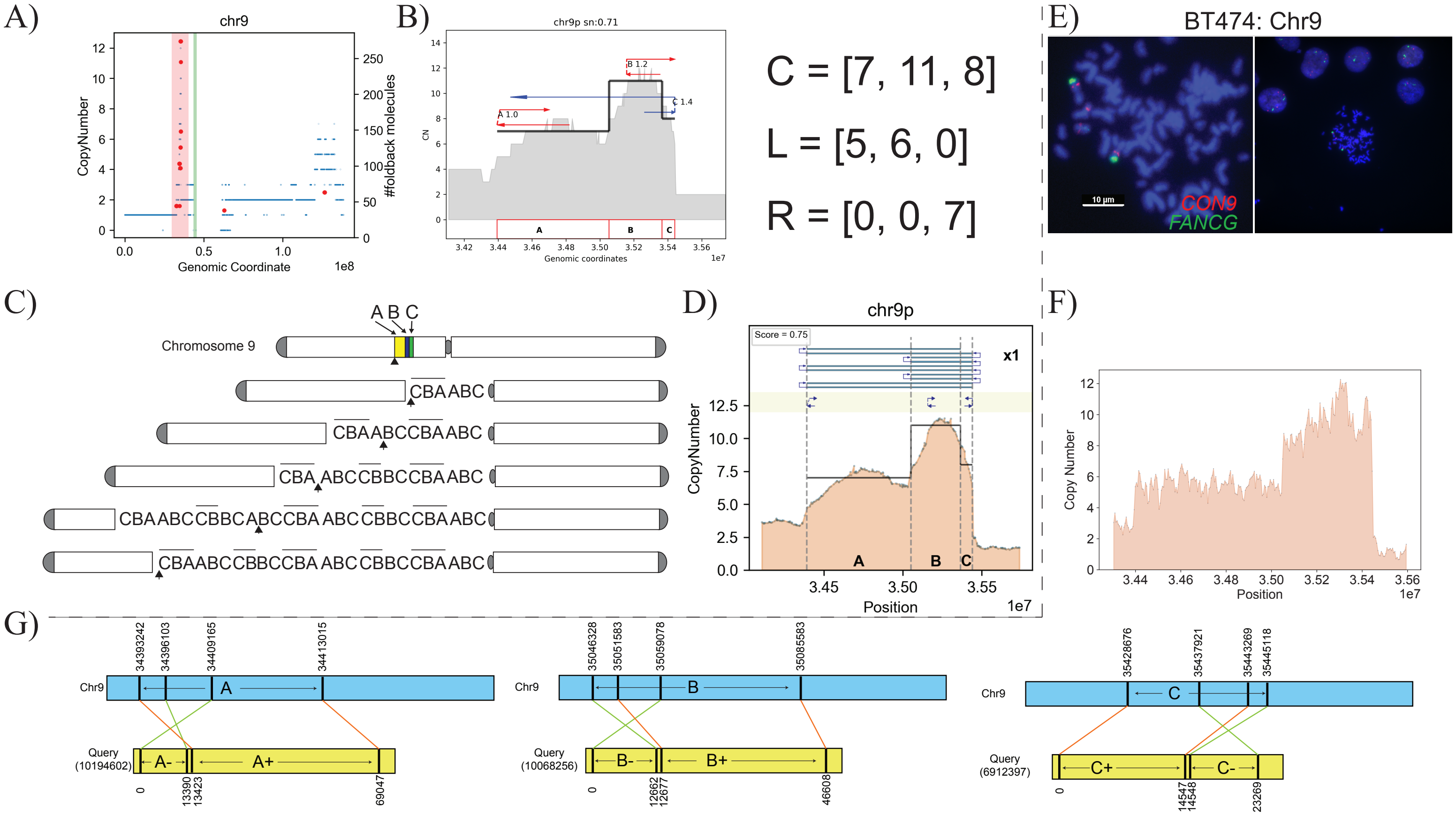

Supplementary Figure 6: Validation of OM2BFB results in BT474 cells on chromosome 9 using DNA Metaphase FISH and Nanopore sequencing. A) Copy number plot for chromosome 9. B) Enlarged view of the copy number plot with foldback reads and copy number vectors indicated. C) Sequential depiction of BFB cycles leading to the final chromosomal structure. D) Final OM2BFB output and predicted BFB structure. E) DNA Metaphase FISH images showing the amplification on the native chromosome 9. F) Copy number plot derived from nanopore sequencing reads. G) Visualization of foldback reads detected by nanopore sequencing

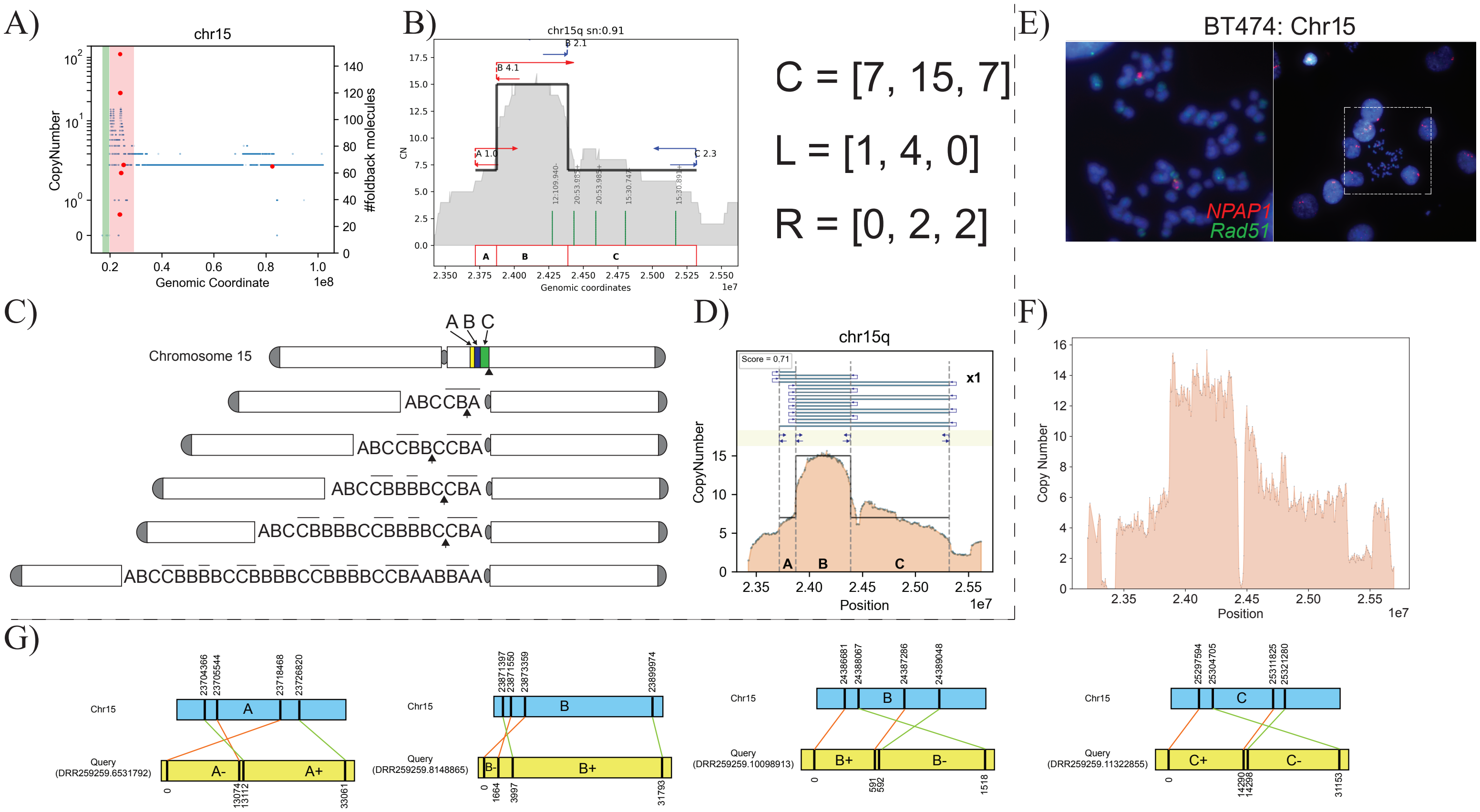

Supplementary Figure 7: Validation of OM2BFB results in BT474 cells on chromosome 15 using DNA Metaphase FISH and Nanopore sequencing. A) Copy number plot for chromosome 15. B) Enlarged view of the copy number plot with foldback reads and copy number vectors indicated. C) Sequential depiction of BFB cycles leading to the final chromosomal structure. D) Final OM2BFB output and predicted BFB structure. E) DNA Metaphase FISH images showing the amplification on the native chromosome 15. F) Copy number plot derived from nanopore sequencing reads. G) Visualization of foldback reads detected by nanopore sequencing.

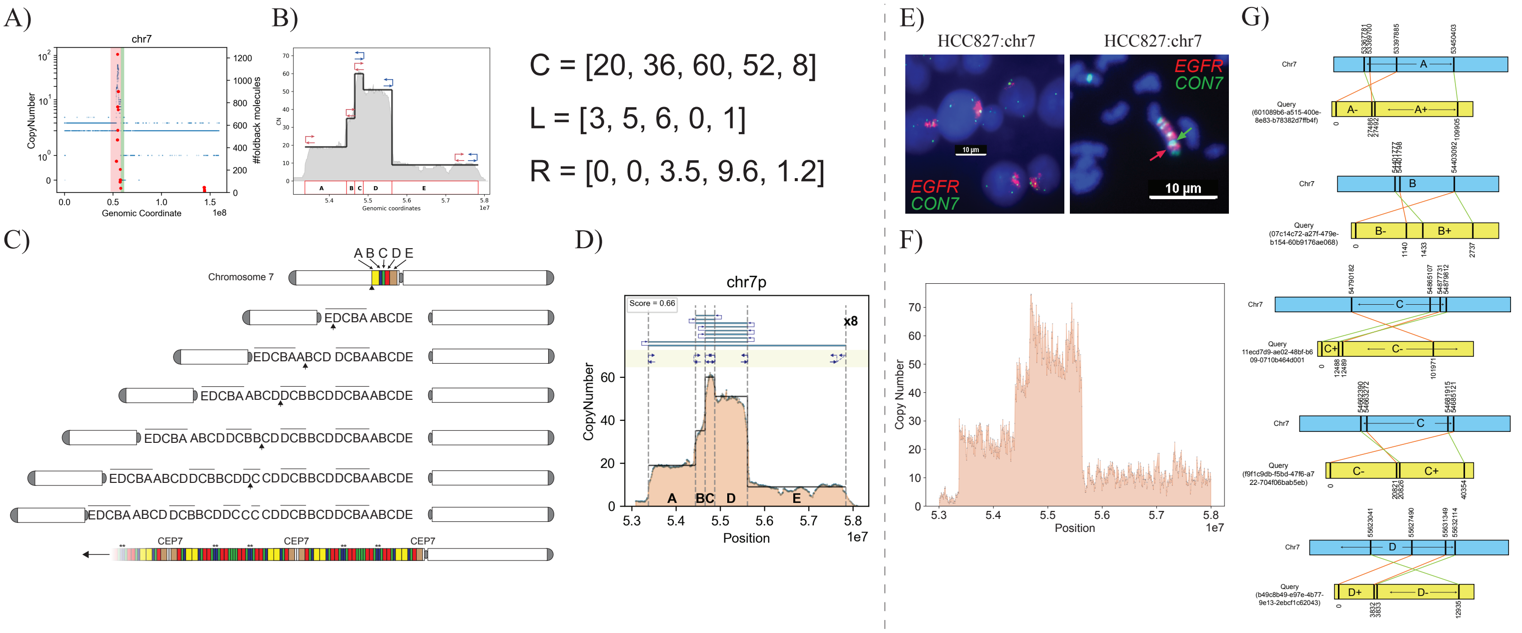

Supplementary Figure 8: Validation of OM2BFB results in HCC827 cells on chromosome 7 using DNA Metaphase FISH and Nanopore sequencing. A) Copy number plot for chromosome 7. B) Enlarged view of the copy number plot with foldback reads and copy number vectors indicated. C) Sequential depiction of BFB cycles leading to the final chromosomal structure. D) Final OM2BFB output and predicted BFB structure. E) DNA Metaphase FISH images showing the amplification on the native chromosome 7. F) Copy number plot derived from nanopore sequencing reads. G) Visualization of foldback reads detected by nanopore sequencing.

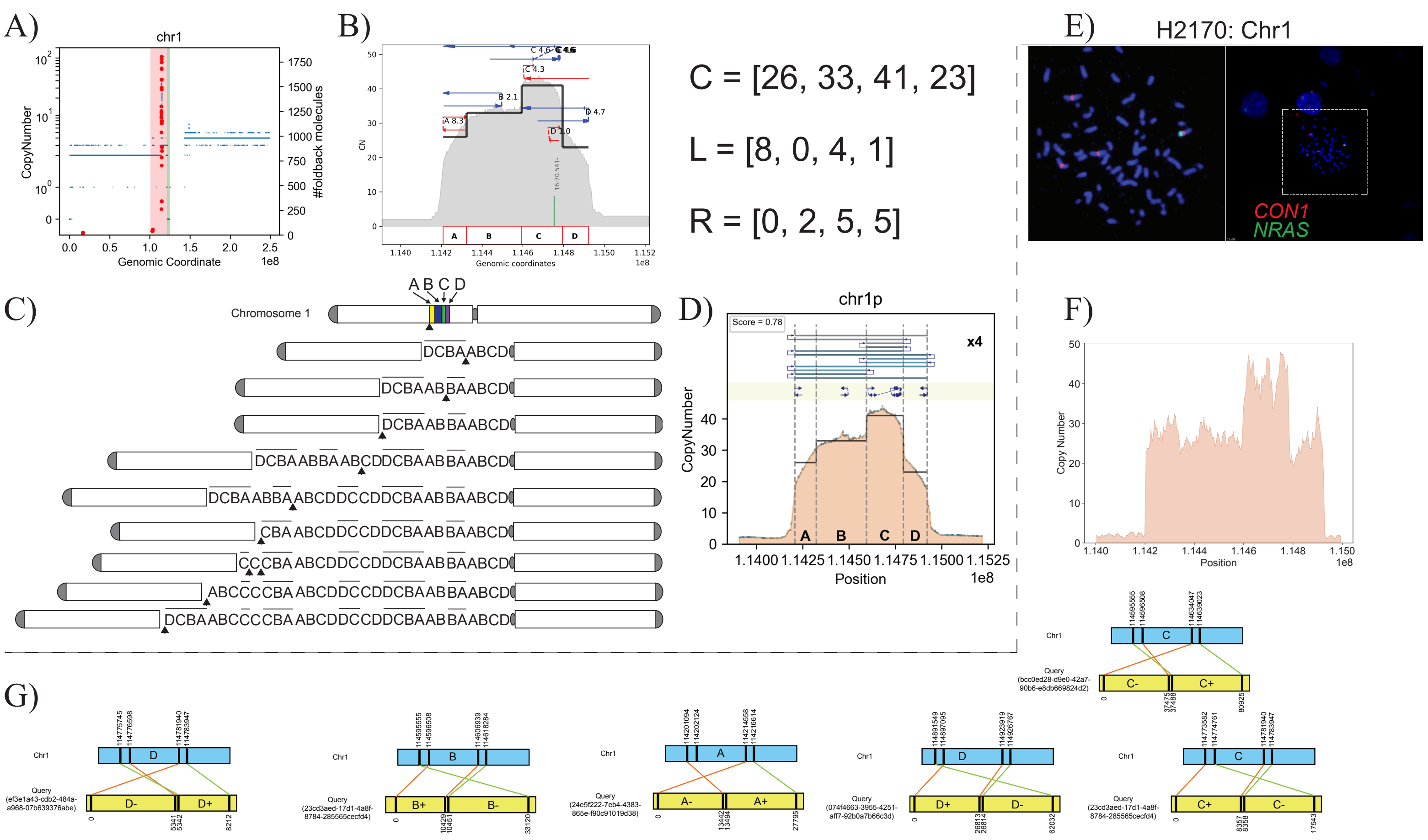

Supplementary Figure 9: Validation of OM2BFB results in H2170 cells on chromosome 1 using DNA Metaphase FISH and Nanopore sequencing. A) Copy number plot for chromosome 1. B) Enlarged view of the copy number plot with foldback reads and copy number vectors indicated. C) Sequential depiction of BFB cycles leading to the final BFB structure. D) Final OM2BFB output and predicted chromosomal structure. E) DNA Metaphase FISH images showing the amplification on the native chromosome 1. F) Copy number plot derived from nanopore sequencing reads. G) Visualization of foldback reads detected by nanopore sequencing.

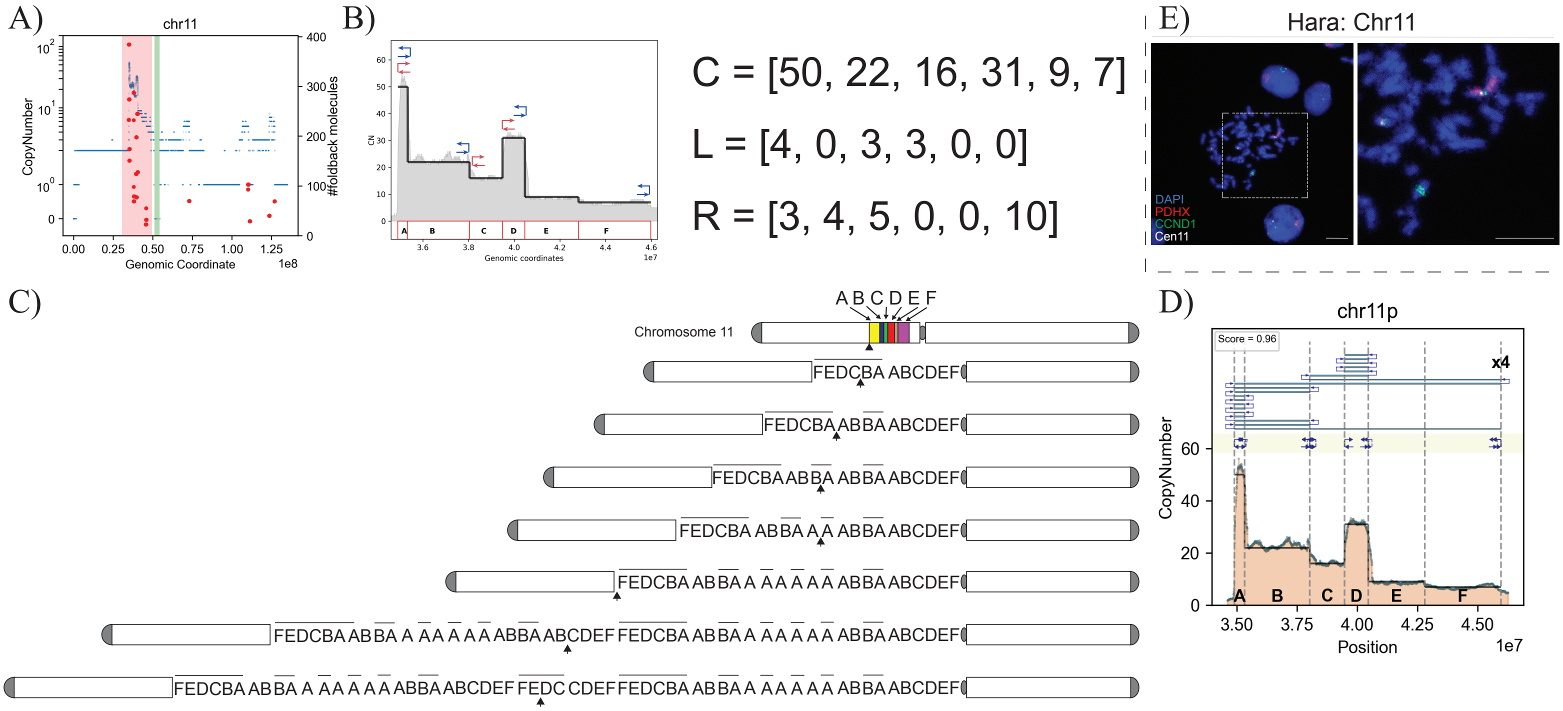

Supplementary Figure 10: Validation of OM2BFB results in Hara cells on chromosome 11 using DNA Metaphase FISH. A) Copy number plot for chromosome 11. B) Enlarged view of the copy number plot with foldback reads and copy number vectors indicated. C) Sequential depiction of BFB cycles leading to the final chromosomal structure. D) Final OM2BFB output and predicted BFB structure. E) DNA Metaphase FISH images showing the amplification on the native chromosome 11.

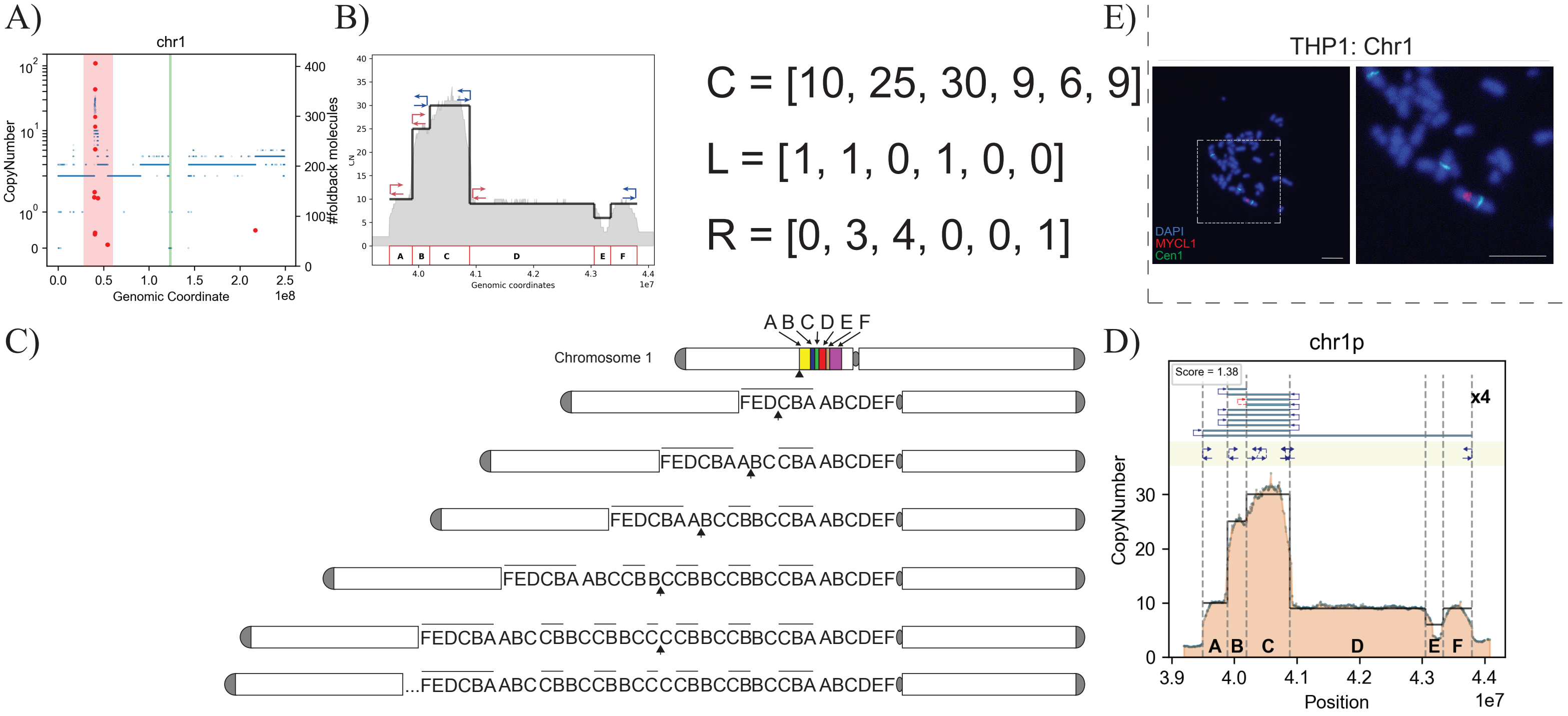

Supplementary Figure 11: Validation of OM2BFB results in THP1 cells on chromosome 1 using DNA Metaphase FISH. A) Copy number plot for chromosome 1. B) Enlarged view of the copy number plot with foldback reads and copy number vectors indicated. C) Sequential depiction of BFB cycles leading to the final chromosomal structure. D) Final OM2BFB output and predicted BFB structure. E) DNA Metaphase FISH images showing the amplification on the native chromosome 1.

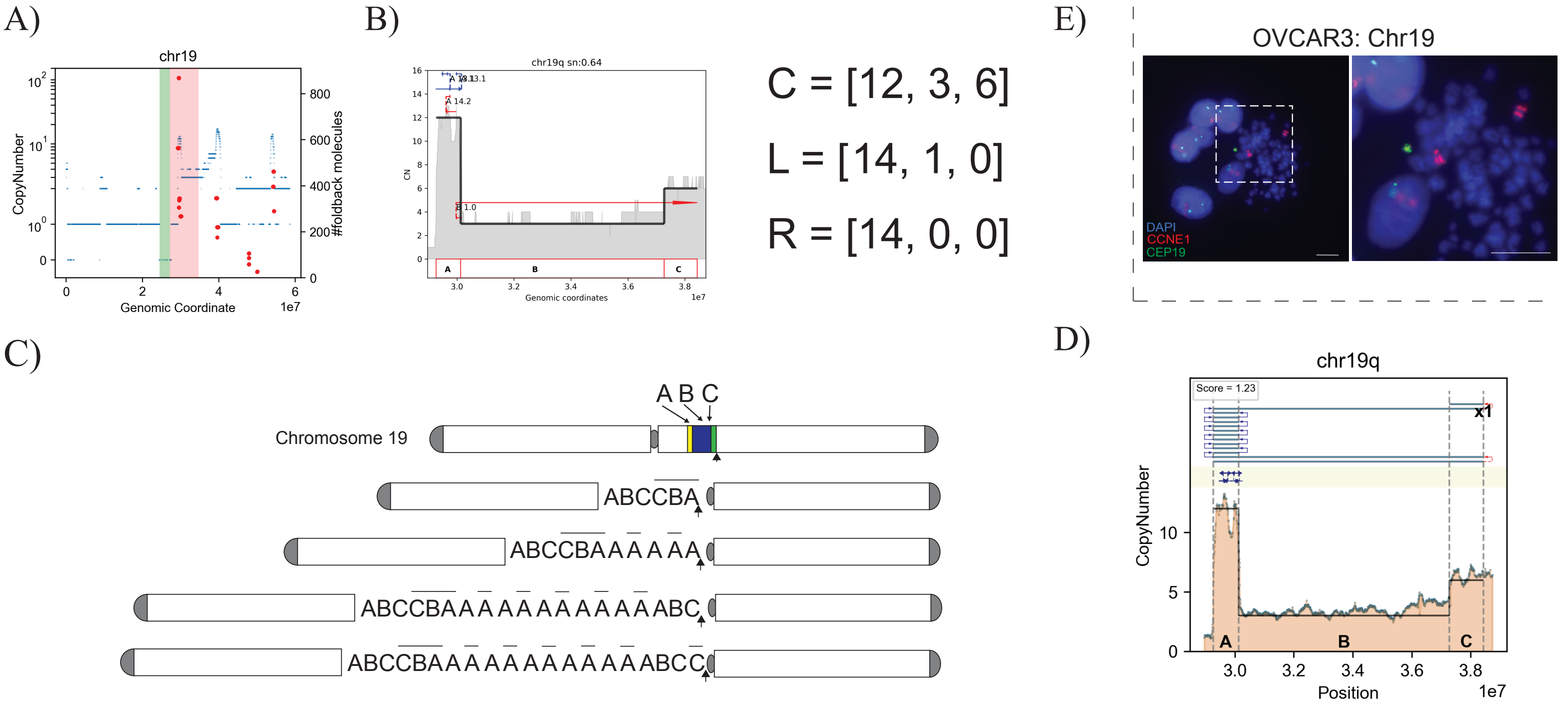

Supplementary Figure 12: Validation of OM2BFB results in OVCAR3 cells on chromosome 19 using DNA Metaphase FISH. A) Copy number plot for chromosome 19. B) Enlarged view of the copy number plot with foldback reads and copy number vectors indicated. C) Sequential depiction of BFB cycles leading to the final chromosomal structure. D) Final OM2BFB output and predicted BFB structure. E) DNA Metaphase FISH images showing the amplification on the native chromosome 19.

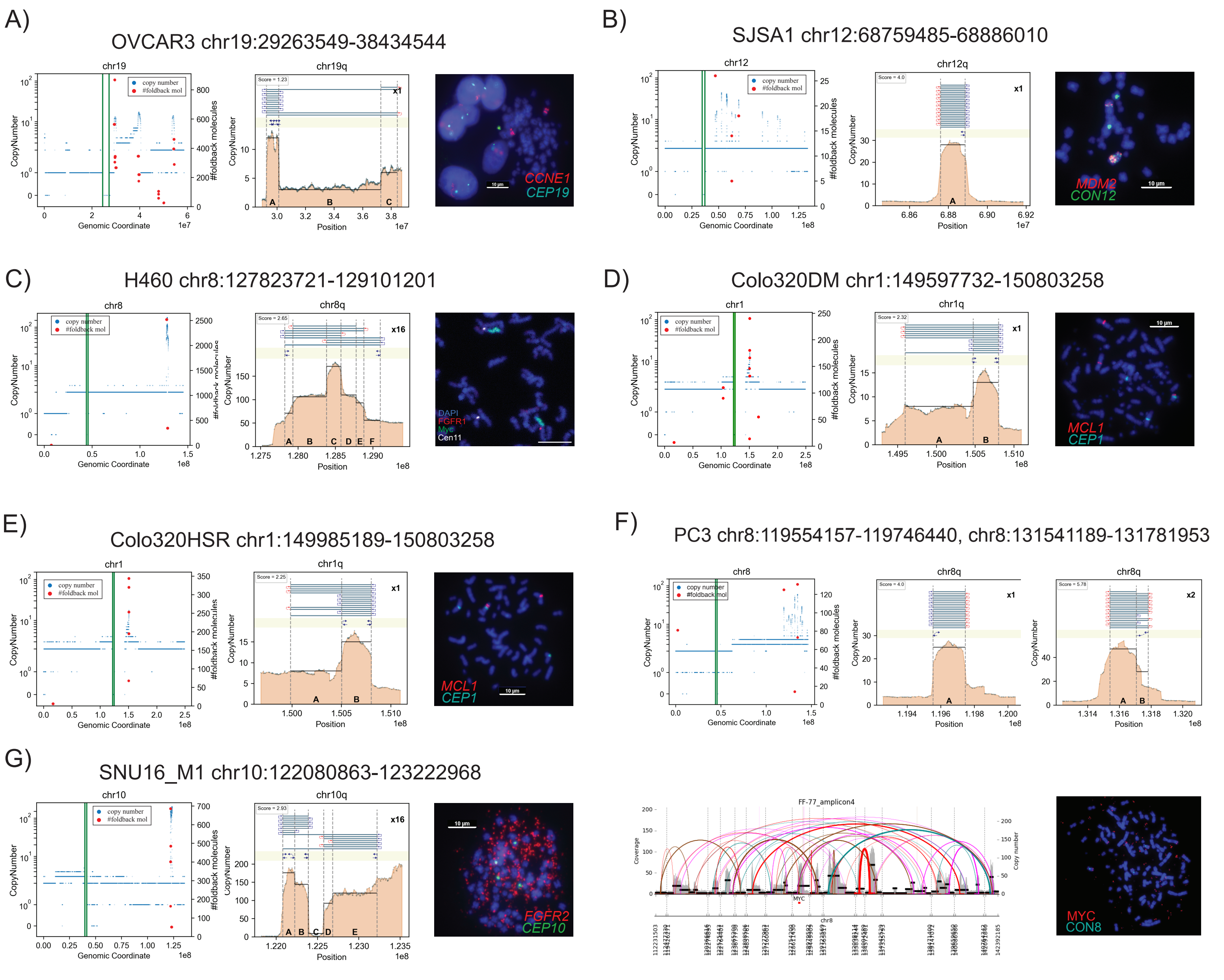

Supplementary Figure 13: Validation of OM2BFB results with DNA Metaphase FISH. (A) A BFB(-) amplification in a non-native chromosome with an OM2BFB score less than 1.8 (false positive call). (B-G) Metaphase FISH images for cancer cell line samples with OM2BFB scores greater than 1.8 showing either HSR formation on non-native chromosomes (panels B-E), or ecDNA (panels F-G). Metaphase FISH images for Colo320DM (chr8 MYC), Colo320HSR (chr8 MYC) and DU145 (chr14 NFKBIA) can be found in the earlier publication.

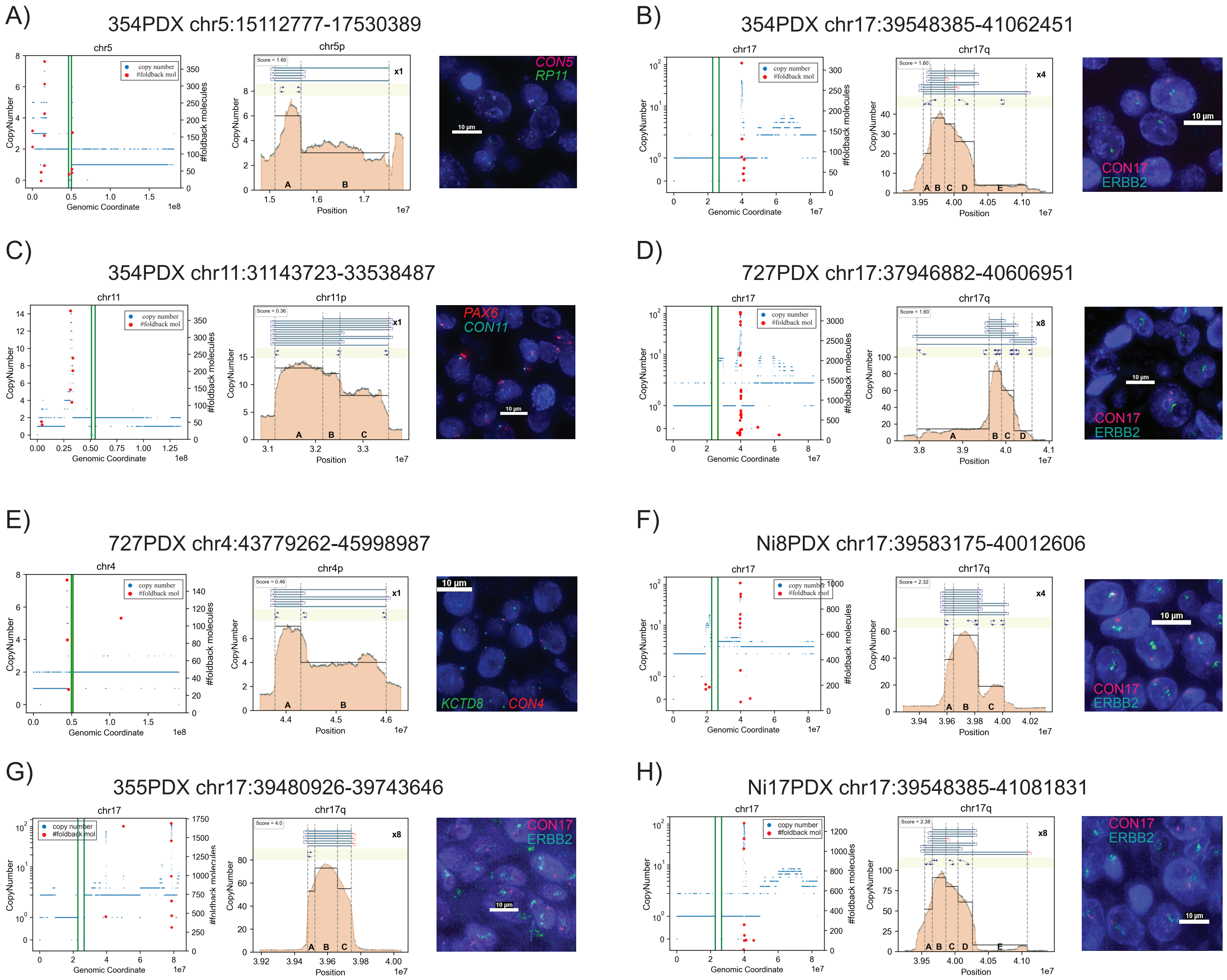

Supplementary Figure 14: Validation of OM2BFB results with DNA Interphase FISH. (A-E) Interphase FISH images for Breast Cancer with Brain Metastases samples with OM2BFB scores lower than 1.8 showing small numbers of distinct foci with low cell to cell heterogeneity. (F-H) Interphase FISH images for Breast Cancer with Brain Metastases samples with OM2BFB scores greater than 1.8 showing larger numbers of distinct foci with high cell to cell heterogeneity.

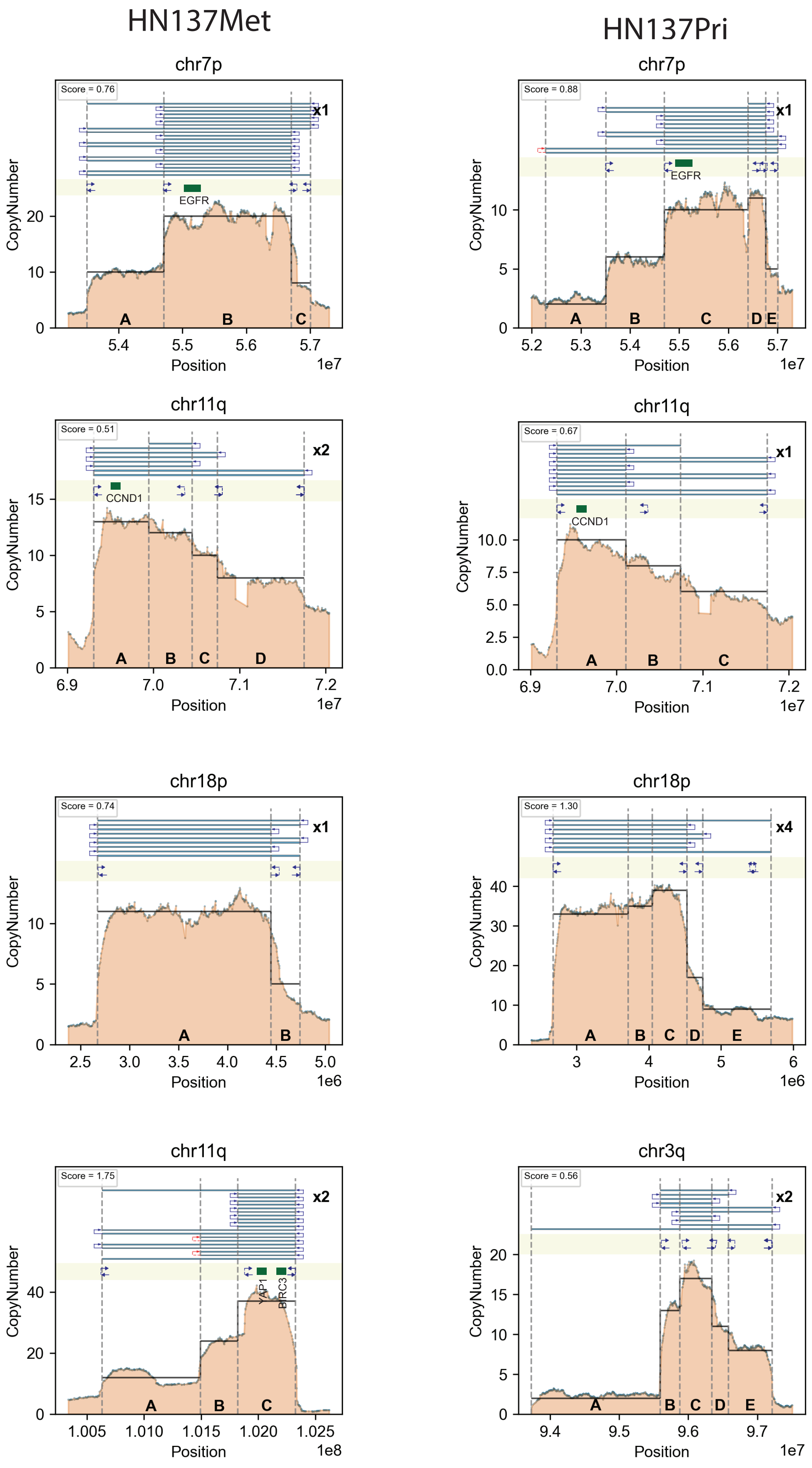

Supplementary Figure 15: BFB amplifications in primary and metastatic cell lines derived from a patient with head and neck cancer.

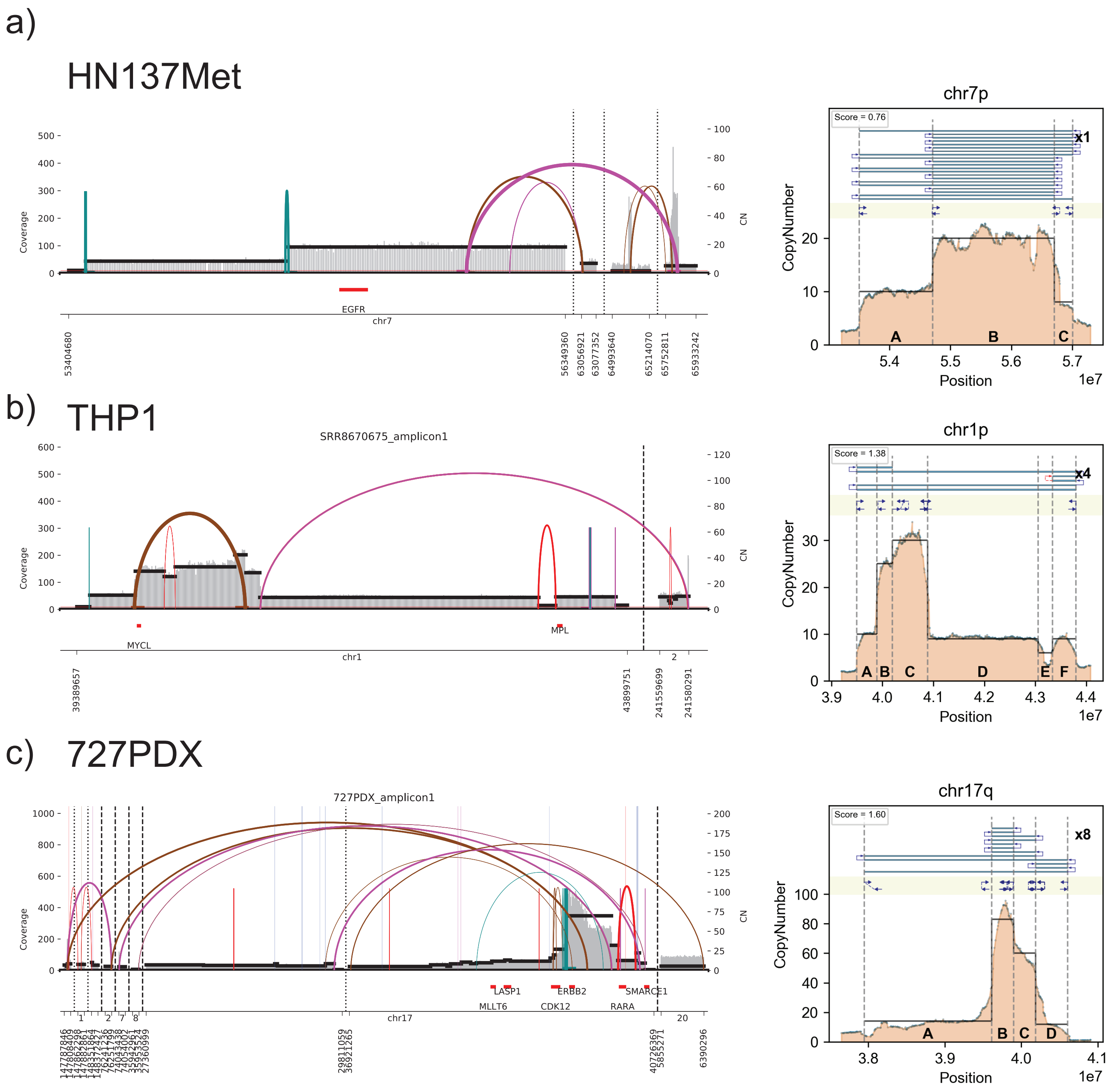

Supplementary Figure 16: Exemplars of discrepancies between OM2BFB predictions on optical genome map data and Amplicon Classifier on whole genome sequencing data. In the discrepant examples, OM2BFB prediction was BFB(+) (Right, score <1.8), while AC prediction was BFB(-) (Left). A, B) Missed foldbacks in wgs data (red dashed); C) Missed foldbacks and the presence of additional translocations lead to a BFB(-) prediction for AC.

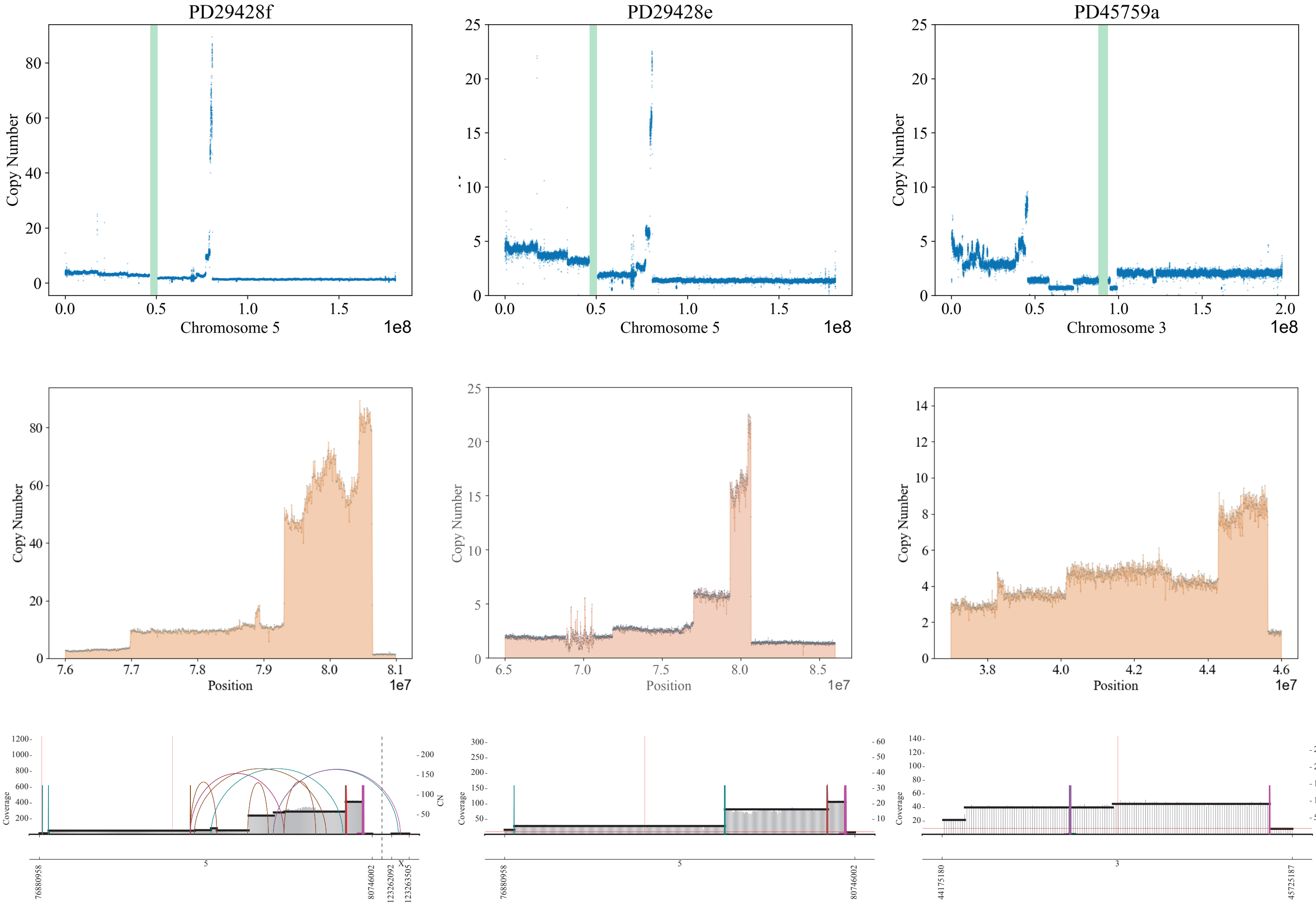

Supplementary Figure 17: Copy number and AA plots for BFB (+) predicted cases from experimental models of BFB23. Amplicon Suite, consisting of Amplicon Architect (AA) and Classifier (AC) predicted BFB cycles in 3 experimentally generated BFB samples.

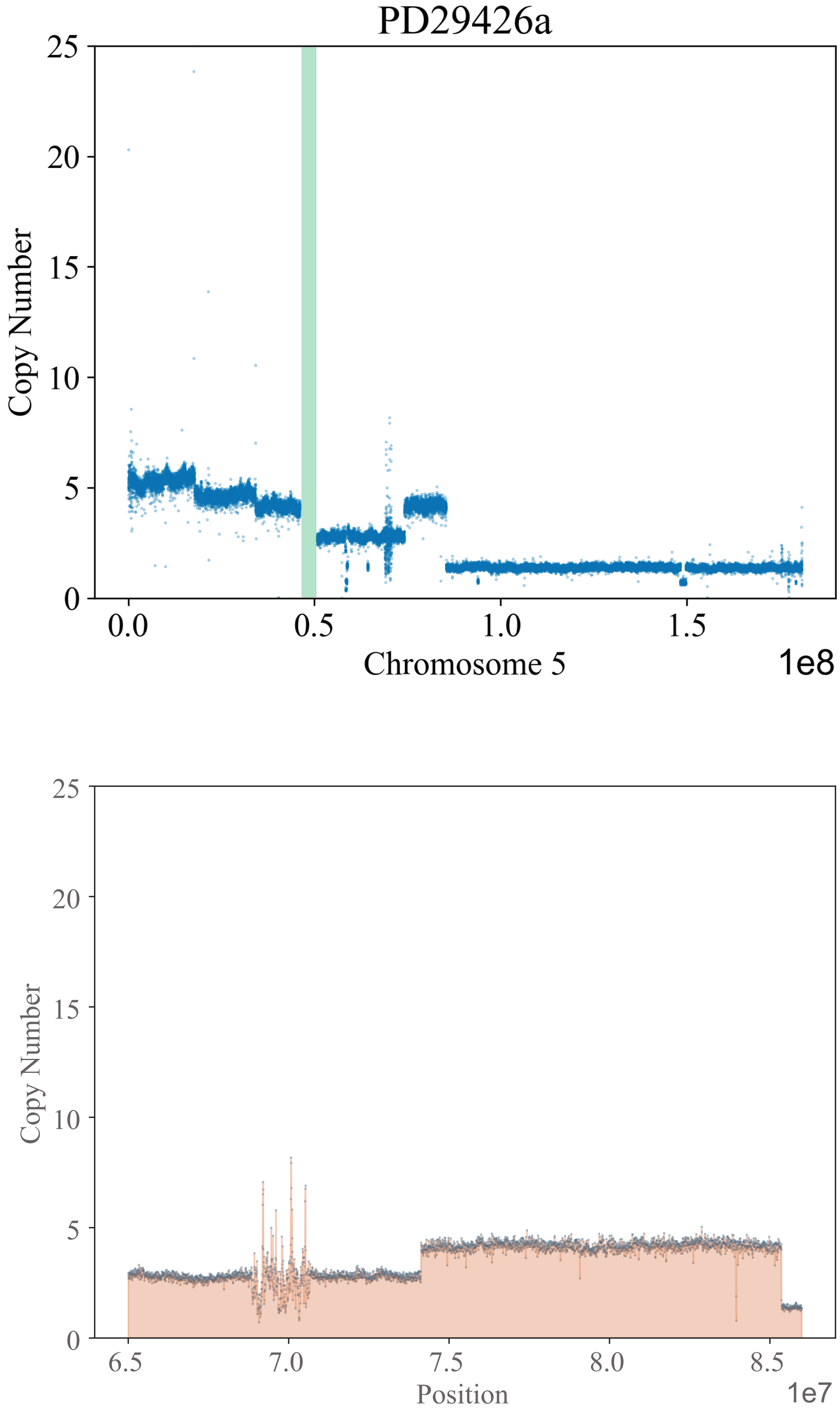

Supplementary Figure 18: Copy number and AA plots for a "No-amp" predicted case from experimental models of BFB. Amplicon Suite, consisting of Amplicon Architect (AA) and Classifier (AC) predicted "no-amp" in an experimentally generated BFB cycle, where the small number of cycles do not show a focal amplification.

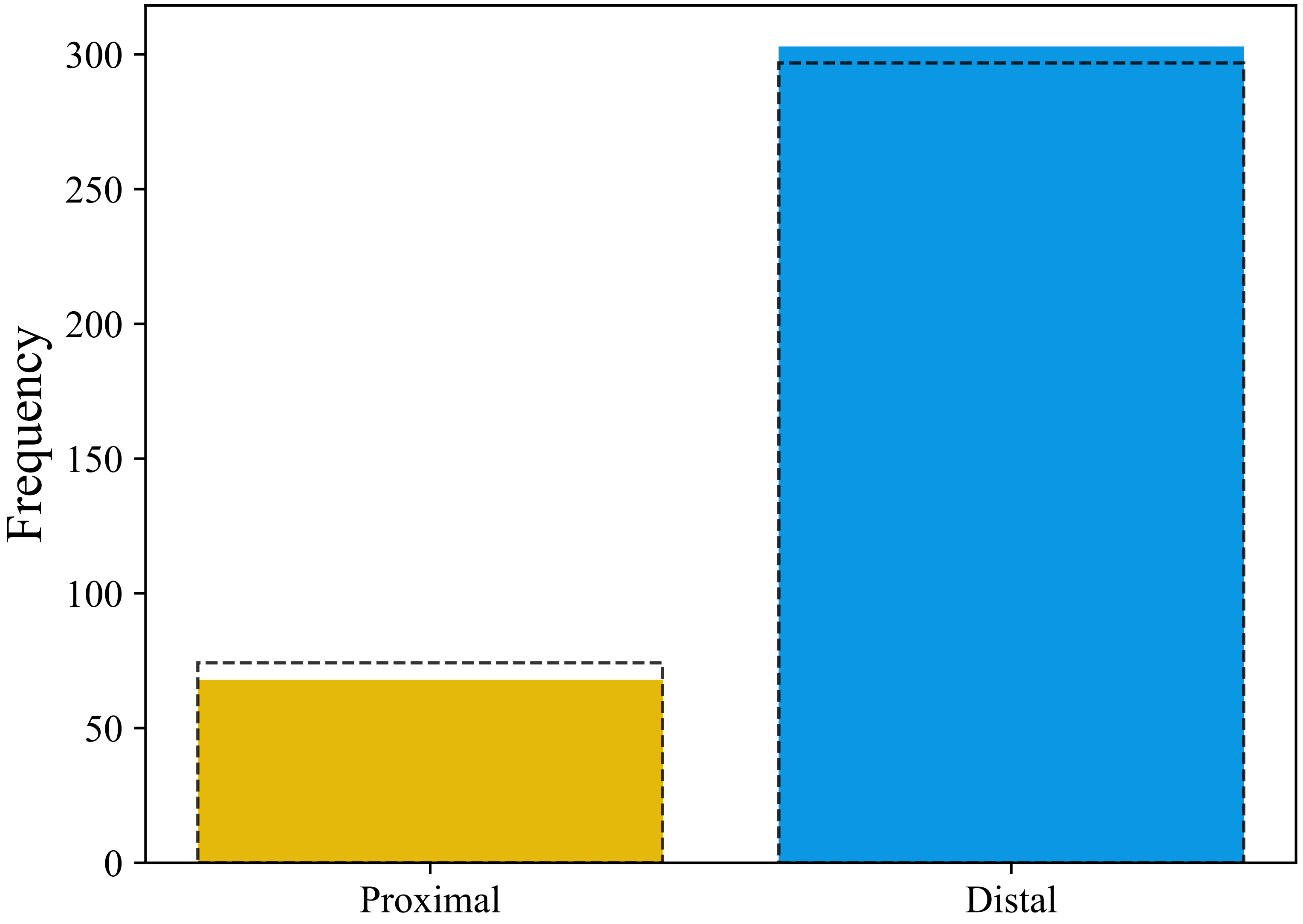

Supplementary Figure 19: Distribution of first break location in proximal/distal regions. Regions comprising 5 percent of chromosome length around each telomere and centromere of every chromosome (20 percent of chromosome length in total) as being “telomeric or centromeric”. BFBs events as being ‘proximal’ if they fell in the telomeric or centromeric window and distal. Out of 371 cases, 68 were found in proximal regions, while 303 were not (two-tailed Binomial test, p-value = 0.48, test statistic = 68). Source data are provided as a Source Data file.

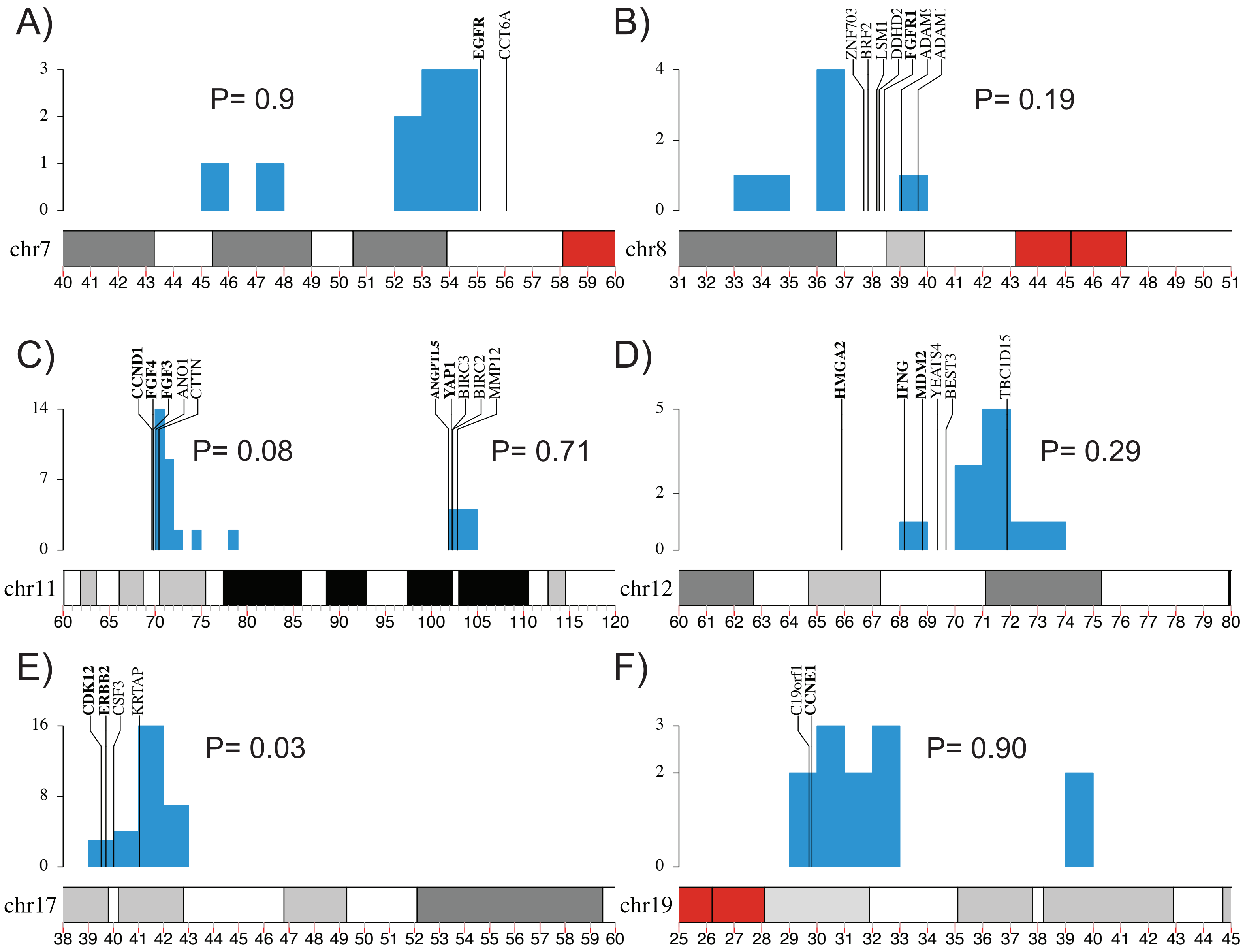

Supplementary Figure 20: Distribution of first-break locations of recurring BFB amplicons. Panels A-F denote 7 cases with multiple BFB amplifications. The distribution of the most telomeric break to an amplified oncogene is plotted in 10 windows, each of 1Mb (grey tick marks). No 1Mb window is preferentially chosen except for the ERBB2 amplified BFB sites, where a preferential break occurs in the KRTAP region. A preferred (but not statistically significant) window is also seen in BFB sites involving the CCND1 oncogene. Source data are provided as a Source Data file.

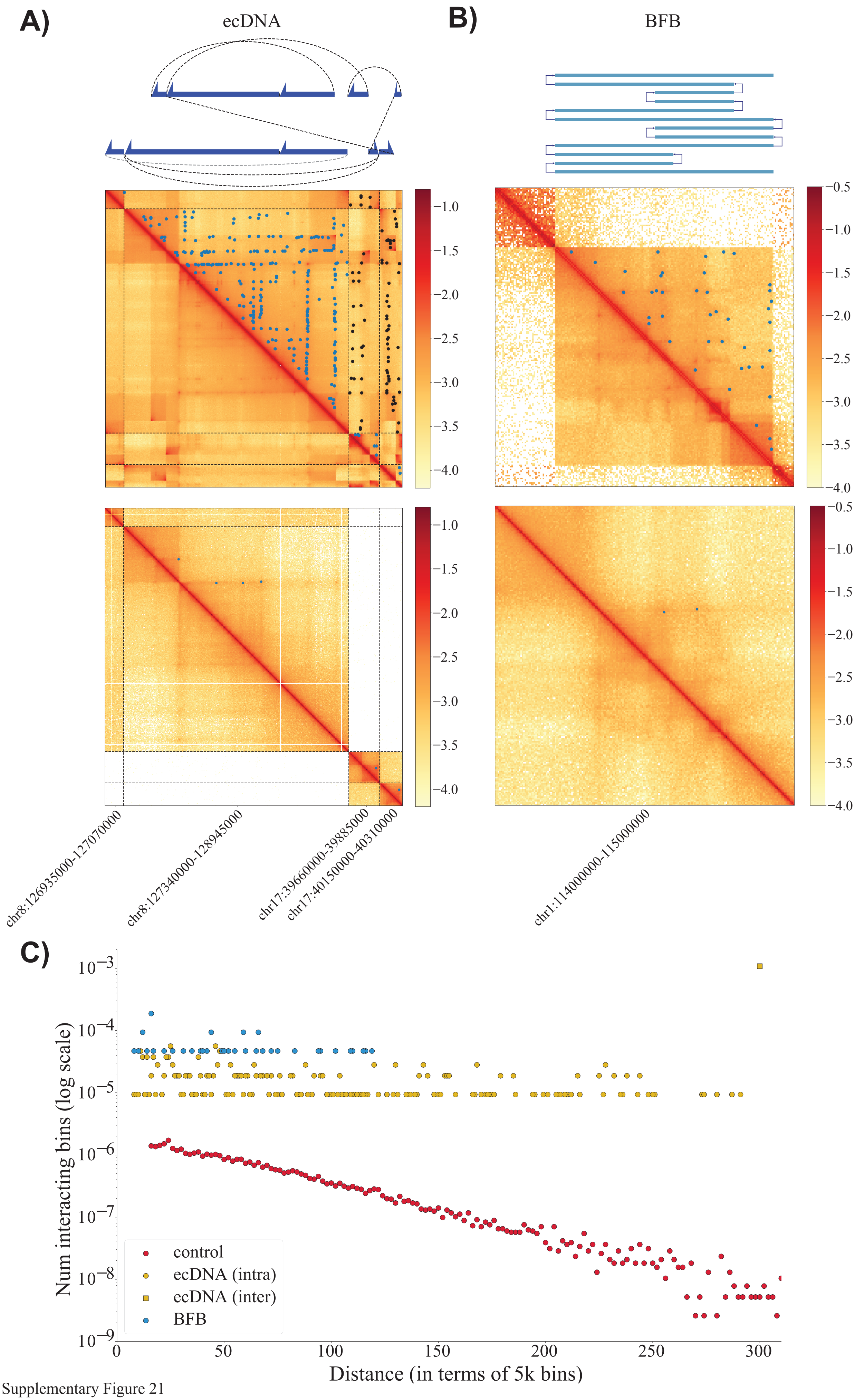

Supplementary Figure 21

Supplementary Figure 21: Chromatin interactions captured by Hi-C in a BFB cycle and ecDNA in the cell-line H2170. A) The structure of ecDNA from the H2170 cell line. Blue arrows indicate genomic segments from chr 8 and 17 amplified on the ecDNA; black dashed lines indicate SV breakpoints directly connecting two remote genomic segments; gray dashed lines indicate templated insertions of segments involving other genomic segments. Normalized Hi-C contact map of H2170 at the ecDNA locus. Colors indicate normalized contact frequencies from the most intensive (red) to the least intensive (yellow). Blue spots indicate significant chromatin interactions identified by NeoLoopFinder; while black spots indicate "neoloops" proximal to SV breakpoints and likely to be formed due to the genomic segments coming together in the cell line. Bottom: Normalized Hi-C contact map of control cell-line GM12878 at the identical chr8 and chr17 locus. Colors indicate normalized contact frequencies from the most intensive (red) to the least intensive (yellow). Blue spots indicate significant chromatin interactions. B) The inferred structure of the BFB-cycle on Chr1 of H2170. Normalized Hi-C contact map of H2170 at the BFB locus. Colors indicate normalized contact frequencies from the most intensive (red) to the least intensive (yellow). Blue spots indicate significant chromatin interactions identified by NeoLoopFinder. Bottom: Normalized Hi-C contact map of GM12878 at the identical chr1 locus. Colors indicate normalized contact frequencies from the most intensive (red) to the least intensive (yellow). Blue spots indicate significant chromatin interactions. C) Distribution of HiC interaction frequencies in ecDNA and BFB-driven amplifications. For a specific genomic distance d (x-axis), the dot represents the fraction, among all pairs of genomic windows separated by d, of pairs with significant Hi-C interactions. Interactions between different chromosomal segments due to ecDNA are shown in the top right. Source data are provided as a Source Data file.

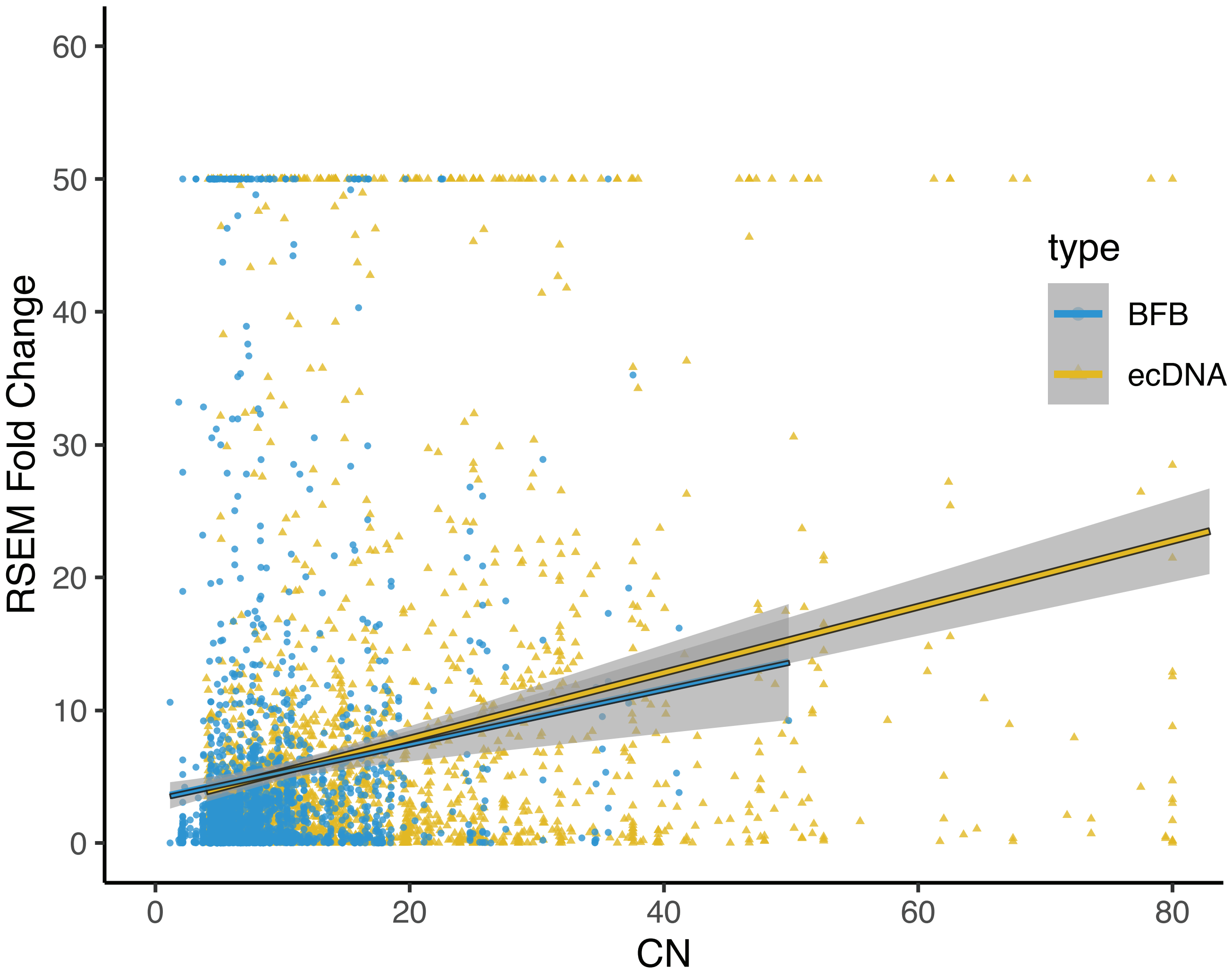

Supplementary Figure 22: Gene expression of oncogenes vs copy number: Copy Number of oncogene versus its fold change in RSEM for oncogenes with copy number >4 in the TCGA data set. Each point represents the average fold change of a specific oncogene across all samples containing this amplified oncogene as BFB or ecDNA. Source data are provided as a Source Data file.

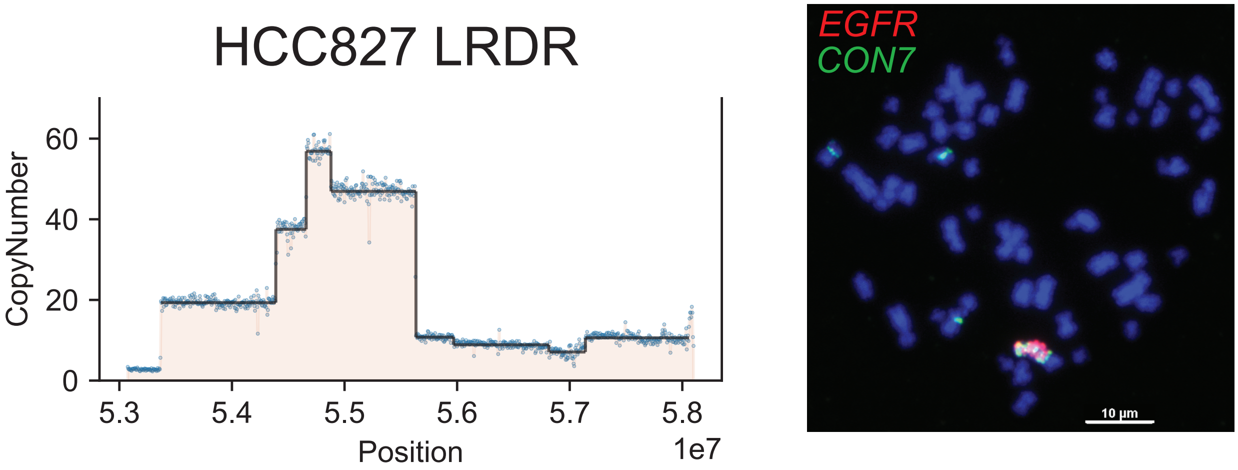

Supplementary Figure 23: HCC827-LRDR. Targeted Therapy Resistance of the HCC827 Cell Line continuing EGFR amplified within a BFB event. The copy number and the proportion of cells carrying the BFB signal are restored after drug removal (LRDR) in the Lapatinib drug resistant cell line along with metaphase FISH images

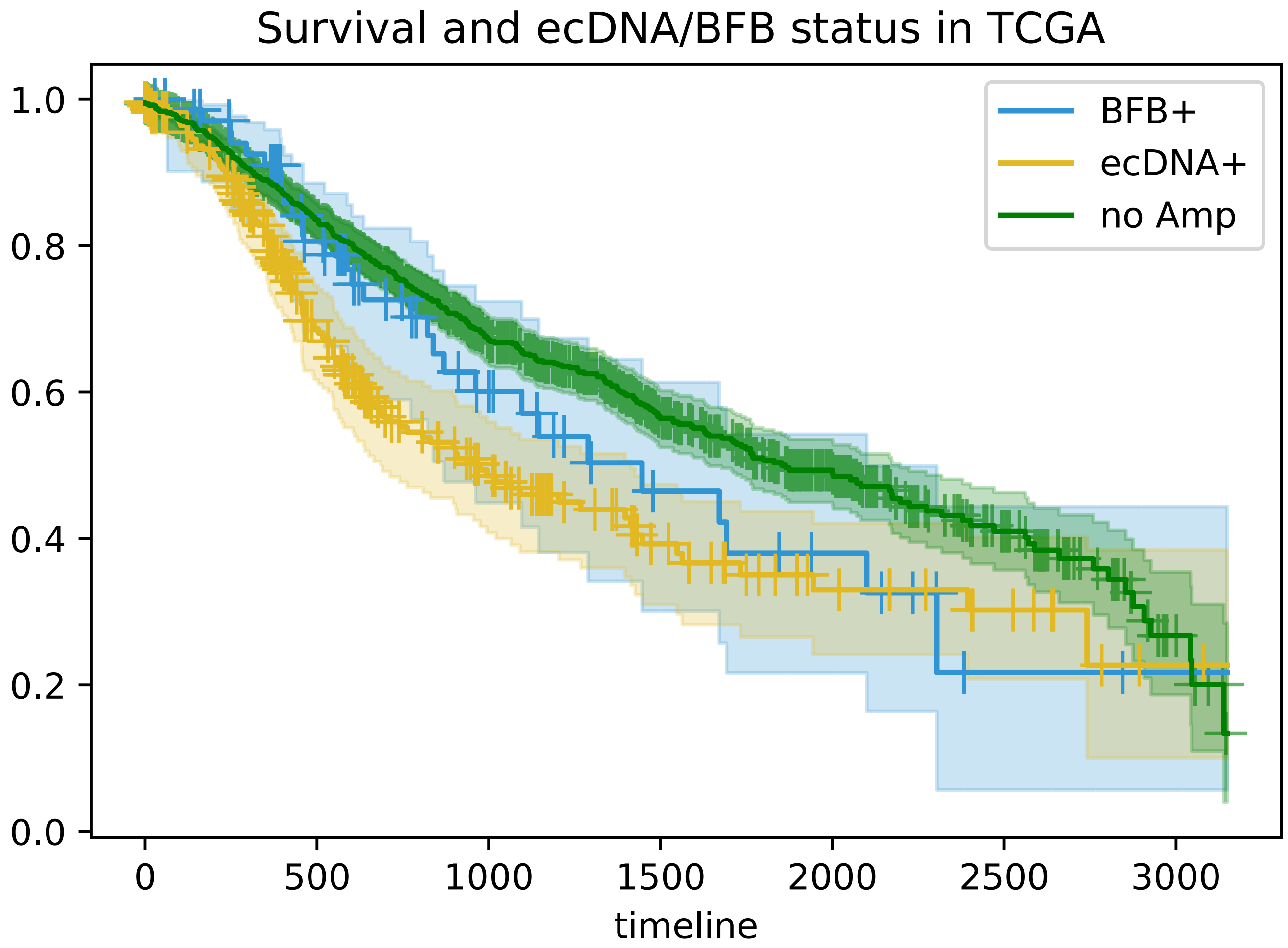

Supplementary Figure 24: Survival rate. The survival outcomes for 71 patients with BFB but no ecDNA amplifications in their tumours compared to outcomes for 231 patients with ecDNA amplifications. BFB(+) individuals have better outcomes initially (first 1100 days). For comparisons, the survival outcome for patients (n=997) with no amplification is also plotted. Patients with survival times exceeding 3000 days were excluded from this analysis. Source data are provided as a Source Data file.

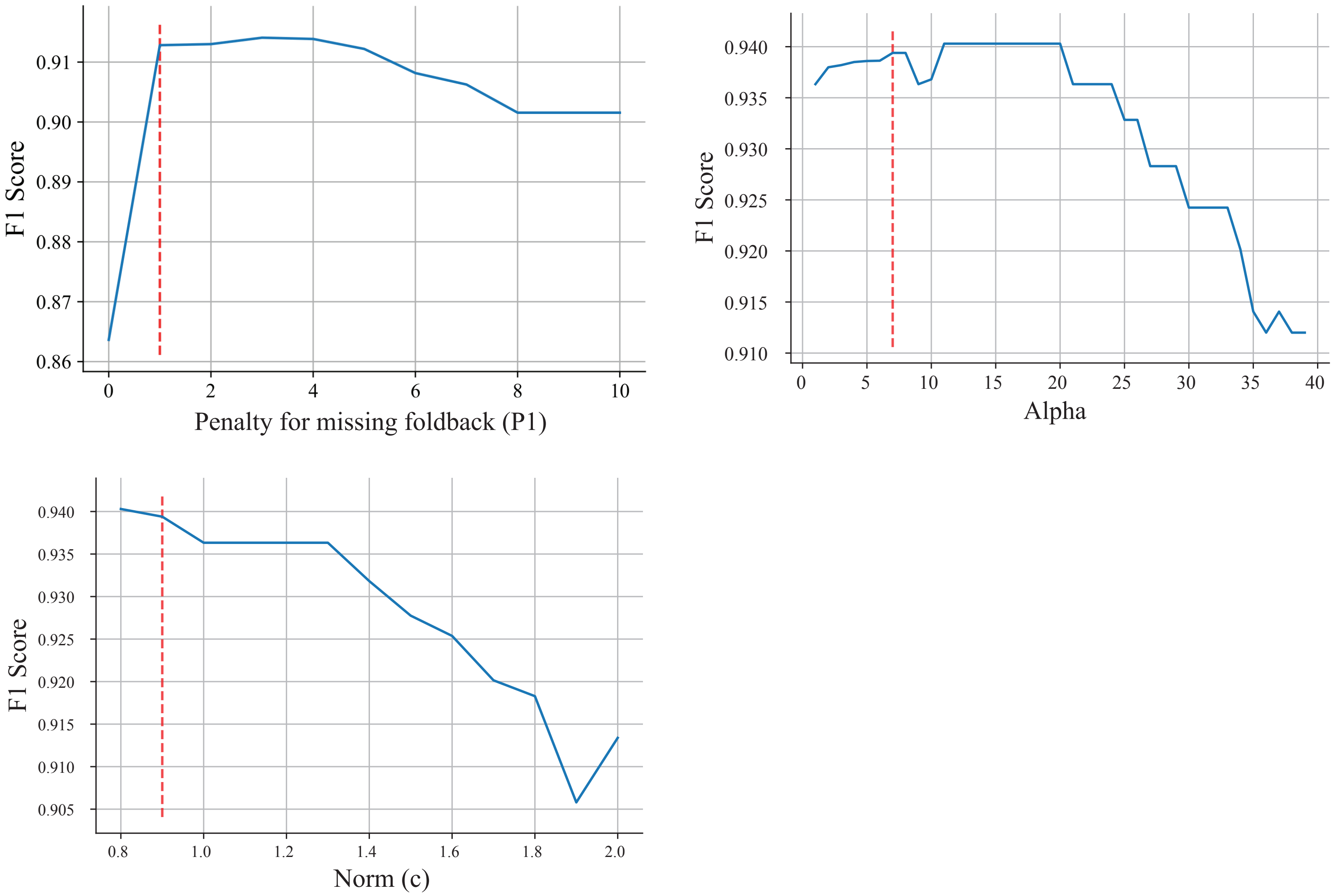

Supplementary Figure 25: Optimizing OM2BFB parameters empirically using BFB (+) samples (n=90) and ecDNA(+) samples (n=90). Source data are provided as a Source Data file.

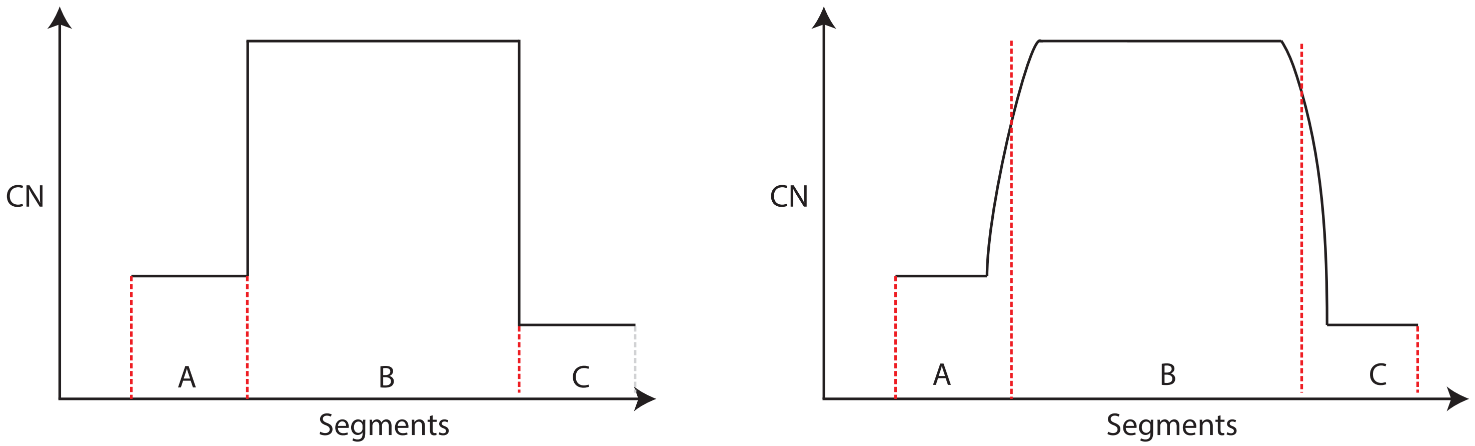

Supplementary Figure 26: CNV call simulation. Illustrations of two different cases of CNV call simulation. The left panel represents segments of CN without any added noise, resulting in clear and distinct boundaries. In contrast, the right panel includes added noise, making the copy number segmentation boundaries more gradual and less defined.

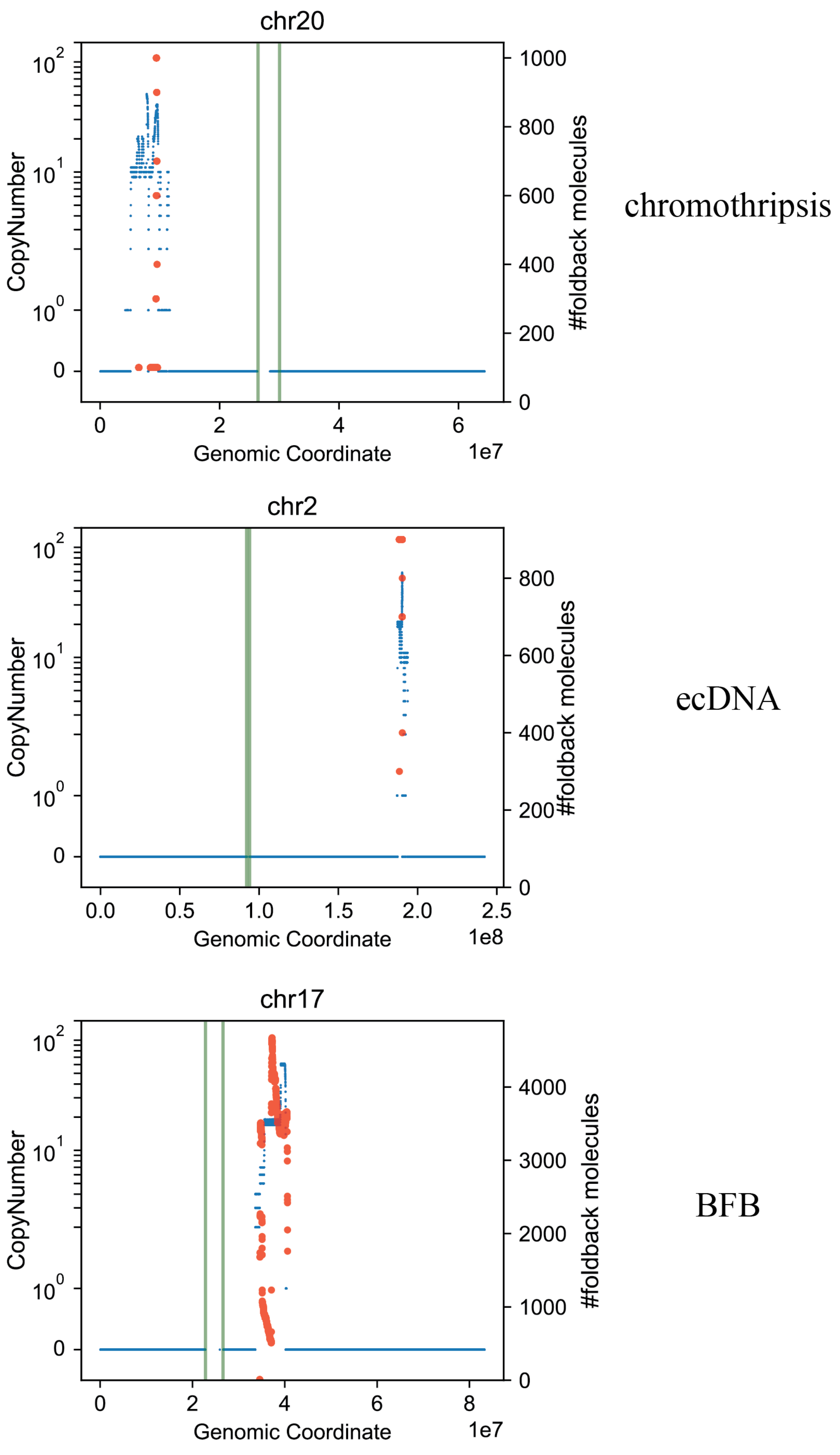

Supplementary Figure 27: Simulated copy number plot for BFB (+), ecDNA(+) and chromothripsis.
