# Supplementary material for: Breakage fusion bridge cycles drive high oncogene number with moderate intratumoural heterogeneity
Source: Nat Commun. 2025 Feb 10;16:1497. doi: 10.1038/s41467-025-56670-8 (PMC11811125; doi:10.1038/s41467-025-56670-8)
Supplement: Supplementary file 3 — Description of Additional Supplementary Files [file 41467_2025_56670_MOESM3_ESM.pdf]

Description of Additional Supplementary Files

Supplementary Data 1: Validation of OM2BFB experiments using metaphase and interphase FISH experiments.

Supplementary Data 2: Validation of AC predictions of BFB cycles using metaphase and interphase FISH experiments.

Supplementary Data 3: Validation of AC predictions of BFB cycles against experimentally generated BFB models.

Supplementary Data 4: Sample analysis of TCGA, CCLE, and BE using Amplicon Classifier.

Supplementary Data 5: Recurrently amplified genomic regions via BFB mechanism at least 7 times with corresponding P-values from BFB distributions calculated with one-tailed permutation-like test.

Supplementary Data 6: Oncogene amplification frequencies via BFB or ecDNA.

Supplementary Data 7: NeoLoopFinder assemblies obtained from Amplicon Architect.

Supplementary Data 8: Exact p-values and statistics for different immune cell subtypes.
