# Supplementary material for: Breakage fusion bridge cycles drive high oncogene number with moderate intratumoural heterogeneity
Source: Nat Commun. 2025 Feb 10;16:1497. doi: 10.1038/s41467-025-56670-8 (PMC11811125; doi:10.1038/s41467-025-56670-8)
Supplement: Supplementary file 5 — Reporting Summary [file 41467_2025_56670_MOESM5_ESM.pdf]

nature portfolio

Corresponding author(s): Vineet Bafna

Last updated by author(s): 2025/01/18

Reporting Summary

Nature Portfolio wishes to improve the reproducibility of the work that we publish. This form provides structure for consistency and transparency in reporting. For further information on Nature Portfolio policies, see our Editorial Policies and the Editorial Policy Checklist.

Statistics

For all statistical analyses, confirm that the following items are present in the figure legend, table legend, main text, or Methods section.

n/a	Confirmed	
☐	☒	The exact sample size (*n*) for each experimental group/condition, given as a discrete number and unit of measurement
☒	☐	A statement on whether measurements were taken from distinct samples or whether the same sample was measured repeatedly
☐	☒	The statistical test(s) used AND whether they are one- or two-sided<br>*Only common tests should be described solely by name; describe more complex techniques in the Methods section.*
☒	☐	A description of all covariates tested
☒	☐	A description of any assumptions or corrections, such as tests of normality and adjustment for multiple comparisons
☐	☒	A full description of the statistical parameters including central tendency (e.g. means) or other basic estimates (e.g. regression coefficient) AND variation (e.g. standard deviation) or associated estimates of uncertainty (e.g. confidence intervals)
☐	☒	For null hypothesis testing, the test statistic (e.g. *F*, *t*, *r*) with confidence intervals, effect sizes, degrees of freedom and *P* value noted<br>*Give P values as exact values whenever suitable.*
☒	☐	For Bayesian analysis, information on the choice of priors and Markov chain Monte Carlo settings
☒	☐	For hierarchical and complex designs, identification of the appropriate level for tests and full reporting of outcomes
☒	☐	Estimates of effect sizes (e.g. Cohen's *d*, Pearson's *r*), indicating how they were calculated

Our web collection on statistics for biologists contains articles on many of the points above.

Software and code

Policy information about availability of computer code

Data collection	Ultra-high molecular weight (UHMW) DNA was isolated from cells using a Bionano Prep SP Blood and Cell Culture DNA Isolation kit (#80042). In brief, about 1 million cells for each sample were lysed and digested in a mixed buffer containing Proteinase K, RNase A, and LBB lysis buffer following the manufacturer's instructions (Bionano Genomics). A Nanobind Disk was then added to the lysate to bind genomic DNA (gDNA) upon the addition of isopropanol. After washing, the gDNA was eluted and subjected to limited shearing to increase homogeneity by slowly pipetting up and down using standard 200 ul tips. The gDNA was then equilibrated overnight at room temperature to enhance homogeneity. 2 ul of gDNA aliquot was diluted in Qubit BR buffer and sonicated for 15 min before measuring concentrations with the Qubit dsDNA BR assay kit (Invitrogen Q3285). The UHMW gDNA was ready for labeling when the coefficient of variation of the Qubit reads were less than 0.3. 750ng purified UHMW DNA was fluorescently labeled at the recognition site CTTAAG with the enzyme DLE-1 and subsequently counter-stained using a Bionano Prep DLS Labeling Kit (#80005) following manufacturer's instructions (Bionano Prep Direct Label and Stain (DLS) Protocol #30206). OGM was performed using a Saphyr platform. Calling of low allele frequency structural variants was performed using the rare variant analysis pipeline (Bionano Solve version 3.6) on molecules ≥ 150kbp in length.<br>AC calls on TCGA were downloaded from Transcriptional immune suppression and upregulation of double stranded DNA damage and repair repertoires in ecDNA-containing tumors, Lin et al. DOI 10.1101/2023.04.24.537925<br>AC calls on BE were downloaded from Extrachromosomal DNA in the cancerous transformation of Barrett's oesophagus, Luebeck et al., Nature 2023<br>AC calls on CCLE were downloaded from amplicon repository.<br>thunder deconvolution version 3.8.2
Data analysis	AmpliconClassifier (AC) version 0.4.11 ((https://github.com/jluebeck/AmpliconClassifier))<br>Bionano Solve version 3.6<br>NeoLoopFinder version 0.4.3

AmpliconArchitect (1.3) (https://github.com/jluebeck/AmpliconArchitect)
Scipy version 1.7.3
CNVKit (version 0.9.7)

For manuscripts utilizing custom algorithms or software that are central to the research but not yet described in published literature, software must be made available to editors and reviewers. We strongly encourage code deposition in a community repository (e.g. GitHub). See the Nature Portfolio guidelines for submitting code & software for further information.

Data

Policy information about availability of data

All manuscripts must include a data availability statement. This statement should provide the following information, where applicable:

- Accession codes, unique identifiers, or web links for publicly available datasets
- A description of any restrictions on data availability
- For clinical datasets or third party data, please ensure that the statement adheres to our policy

The genomic data utilized in this study is sourced from various repositories and studies, adhering to the principles of open science and data sharing. The OGM data from BCBM samples (Methods) is available at PRJNA1022500 [https://www.ncbi.nlm.nih.gov/bioproject/PRJNA1022500/]. All procedures were approved by Dana-Farber Cancer Institute Animal Care and Use Committee. OGM data from Head and Neck Cancer cell-lines HN137Pri and HN137Met is available at PRJNA1022500 [https://www.ncbi.nlm.nih.gov/bioproject/PRJNA1022500/]. All procedures were approved by Singhealth Centralized Institutional Review Board (CIRB 2007/441/B). OGM data for Medulloblastoma cell-lines was downloaded from PRJNA1011359 [https://www.ncbi.nlm.nih.gov/bioproject/PRJNA1011359] as reported in this study [https://doi.org/10.1038/s41588-023-01551-3]. OGM data for cancer cell-lines was acquired and is accessible at PRJNA1022500 [https://www.ncbi.nlm.nih.gov/bioproject/PRJNA1022500/]. WGS data for HCC827, HCC827LR, and HCC827DR are available under accession number PRJNA338012 [https://www.ncbi.nlm.nih.gov/bioproject/PRJNA338012/] from a previous publication [https://doi.org/10.1038/nature21356]. The HCC827-ER WGS is available from SRR31728042 [https://www.ncbi.nlm.nih.gov/sra/SRX27090373]. Amplicon Architect output for HCC827 supporting Figure 4 are publicly available at https://ampliconrepository.org/project/673e3bd2642565afc9a37c56. AmpliconArchitect outputs for TCGA were obtained from a previous study [https://doi.org/10.1038/s41588-020-0678-2] and they are publicly available at https://ampliconrepository.org/project/655bda68bba7c92509522479. AmpliconClassifier (AC) calls on CCLE data were downloaded from https://ampliconrepository.org/project/6580f373ea940f33361428ba. AmpliconArchitect outputs for the Barrett's esophagus/esophageal cancer dataset were obtained from previously published study [https://doi.org/10.6084/m9.figshare.21893826.v1]. Source data are provided with this paper.

Research involving human participants, their data, or biological material

Policy information about studies with human participants or human data. See also policy information about sex, gender (identity/presentation), and sexual orientation and race, ethnicity and racism.

Reporting on sex and gender	Sex and gender data was not collected in this study.
Reporting on race, ethnicity, or other socially relevant groupings	Race and ethnicity data was not collected in this study.
Population characteristics	Population characteristic for BCBM samples: Patient-derived xenografts were derived from fresh breast cancer brain metastases (BCBM) acquired from patients undergoing neurosurgery at the Brigham and Women's Hospital .<br>Population characteristic for Head and Neck samples(HN137-Pri and HN137-Met): Tumour samples were obtained from patients post surgery after obtaining informed patient consent in accordance to SingHealth Centralized Institutional Review Board (CIRB: 2014/2093/B).<br>We did not perform population characteristic analysis on the 23 remaining samples we analyzed with OGM.<br>Population characteristic for TCGA: TCGA: The Cancer Genome Atlas Research Network, Nature 2017<br>Population characteristic for BE: Extrachromosomal DNA in the cancerous transformation of Barrett’s oesophagus, Luebeck et al., Nature 2023<br>Population characteristic for CCLE: The Cancer Cell Line Encyclopedia enables predictive modelling of anticancer drug sensitivity, Barretina et al., Nature 2012
Recruitment	Informed consent was obtained from breast cancer patients and fresh brain metastases were then acquired from patients undergoing neurosurgery at the Brigham and Women's Hospital<br>For Head and Neck samples(HN137-Pri and HN137-Met): Tumour samples were obtained from patients post surgery after obtaining informed patient consent in accordance to SingHealth Centralized Institutional Review Board (CIRB: 2014/2093/B)<br>We did not perform Recruitment analysis on the 23 remaining samples we analyzed with OGM.<br>Recruitment for TCGA: TCGA: The Cancer Genome Atlas Research Network, Nature 2017<br>Recruitment for BE: Extrachromosomal DNA in the cancerous transformation of Barrett’s oesophagus, Luebeck et al., Nature 2023<br>Recruitment for CCLE: The Cancer Cell Line Encyclopedia enables predictive modelling of anticancer drug sensitivity, Barretina et al., Nature 2012
Ethics oversight	For BCBM samples: Acquisition of human samples was approved by the Institutional Review Board (IRB) protocols (DFCI IRB 93-085, 10-417, 18-296)<br>For Head and Neck Samples: The procedures were approved by the Singhealth Centralized Institutional Review Board (CIRB 2007/441/B)<br>We did not perform Ethics oversight analysis on the 23 remaining samples we analyzed with OGM.<br>Ethics oversight for TCGA: TCGA: The Cancer Genome Atlas Research Network, Nature 2017

Ethics oversight for BE: Extrachromosomal DNA in the cancerous transformation of Barrett's oesophagus, Luebeck et al., Nature 2023
Ethics oversight for CCLE: The Cancer Cell Line Encyclopedia enables predictive modelling of anticancer drug sensitivity, Barretina et al., Nature 2012

Note that full information on the approval of the study protocol must also be provided in the manuscript.

Field-specific reporting

Please select the one below that is the best fit for your research. If you are not sure, read the appropriate sections before making your selection.

☒ Life sciences ☐ Behavioural & social sciences ☐ Ecological, evolutionary & environmental sciences

For a reference copy of the document with all sections, see nature.com/documents/nr-reporting-summary-flat.pdf

Life sciences study design

All studies must disclose on these points even when the disclosure is negative.

Sample size	We analyzed 31 samples with OGM.<br>We also analyzed 1538 number of TCGA samples, 270 number of CCLE samples and 305 number of BE samples.
Data exclusions	No data was excluded.
Replication	Replication is not applicable for OGM and WGS data. For each samples we collected at least 8 number of Fish images.
Randomization	randomization is not applicable.
Blinding	All individuals performing whole-genome sequencing analysis were blinded to cancer outcome.

Reporting for specific materials, systems and methods

We require information from authors about some types of materials, experimental systems and methods used in many studies. Here, indicate whether each material, system or method listed is relevant to your study. If you are not sure if a list item applies to your research, read the appropriate section before selecting a response.

Materials & experimental systems

n/a	Involved in the study	
☒	☐	Antibodies
☐	☒	Eukaryotic cell lines
☒	☐	Palaeontology and archaeology
☐	☒	Animals and other organisms
☒	☐	Clinical data
☒	☐	Dual use research of concern
☒	☐	Plants

Methods

n/a	Involved in the study	
☒	☐	ChIP-seq
☒	☐	Flow cytometry
☒	☐	MRI-based neuroimaging

Eukaryotic cell lines

Policy information about cell lines and Sex and Gender in Research

Cell line source(s)	Colo320DM, Colo320HSR, BT474, HARA, H460, HCC827, OVCAR3, SJSA1 and THP1 were obtained from ATCC and Sekisui Xenotech. The monoclonal SNU16m1 was a subline of the parental SNU16 cells obtained from ATCC, generated from a single cell after lentiviral transduction and stable expression of dCas9-KRAB as previously described (PMID: 34819668). HCC827 naive, drug resistant (HCC827 ER, HCC827 LR) and drug removed lines (HCC827 ERDR) were generated by Frank Furnari group.
Authentication	Cell lines purchased from ATCC and Sekisui Xenotech were not authenticated.
Mycoplasma contamination	All cell lines were tested negative for mycoplasma.
Commonly misidentified lines<br>(See ICLAC register)	-

Animals and other research organisms

Policy information about studies involving animals; ARRIVE guidelines recommended for reporting animal research, and Sex and Gender in Research

Laboratory animals	Female ICR-SCID mice (Taconic, IcrTac:ICR-Prkdcscid) aged 6-10 weeks old were used.
Wild animals	No wild animals were used in this study.
Reporting on sex	We only used female mice in this study.
Field-collected samples	no field collections were used in this study.
Ethics oversight	All the animal experiments were performed according to protocols approved by the Dana-Farber Cancer Institute Animal Care and Use Committee in compliance with NIH animal guidelines.

Note that full information on the approval of the study protocol must also be provided in the manuscript.
